# Integrated analysis of transcriptomic and proteomic analyses reveals different metabolic patterns in the livers of Tibetan and Yorkshire pigs

**DOI:** 10.5713/ajas.20.0342

**Published:** 2020-10-13

**Authors:** Mengqi Duan, Zhenmei Wang, Xinying Guo, Kejun Wang, Siyuan Liu, Bo Zhang, Peng Shang

**Affiliations:** 1 College of Animal Science, Tibet Agriculture and Animal Husbandry University, Linzhi, Xizang 86000, China; 2 College of Animal Sciences and Veterinary Medicine, Henan Agricultural University, Zhengzhou, Henan 450046, China; 3 National Engineering Laboratory for Animal Breeding/Beijing Key Laboratory for Animal Genetic Improvement, China Agricultural University, Beijing 100193, China

**Keywords:** Hypoxia, Metabolic, Proteome, Tibetan Pig, Transcriptome

## Abstract

**Objective:**

Tibetan pigs, predominantly originating from the Tibetan Plateau, have been subjected to long-term natural selection in an extreme environment. To characterize the metabolic adaptations to hypoxic conditions, transcriptomic and proteomic expression patterns in the livers of Tibetan and Yorkshire pigs were compared.

**Methods:**

RNA and protein were extracted from liver tissue of Tibetan and Yorkshire pigs (n = 3, each). Differentially expressed genes and proteins were subjected to gene ontology and Kyoto encyclopedia of genes and genomes functional enrichment analyses.

**Results:**

In the RNA-Seq and isobaric tags for relative and absolute quantitation analyses, a total of 18,791 genes and 3,390 proteins were detected and compared. Of these, 273 and 257 differentially expressed genes and proteins were identified. Evidence from functional enrichment analysis showed that many genes were involved in metabolic processes. The combined transcriptomic and proteomic analyses revealed that small molecular biosynthesis, metabolic processes, and organic hydroxyl compound metabolic processes were the major processes operating differently in the two breeds. The important genes include retinol dehydrogenase 16, adenine phosphoribosyltransferase, prenylcysteine oxidase 1, sorbin and SH3 domain containing 2, *ENSSSCG00000036224*, perilipin 2, ladinin 1, kynurenine aminotransferase 1, and dimethylarginine dimethylaminohydrolase 1.

**Conclusion:**

The findings of this study provide novel insight into the high-altitude metabolic adaptation of Tibetan pigs.

## INTRODUCTION

Tibetan pigs, mainly originating from the Tibetan Plateau, have endured long-term natural selection in an extreme environment, such as hypoxia, low temperature, and high solar radiation [1,2]. Adaptation to the extreme environment is complex and reflected in several aspects, including cardiopulmonary function, fat metabolism, immunity, blood pressure, and coat color [3–5]. Some studies have been conducted on hypoxia adaptation, physiological features, meat quality, and animal origin [6–8] of Tibetan pigs. Liver metabolic processes are also an important adaptive feature, as they play a key role in catalytic decomposition, biosynthesis, and energy delivery. Characterization of mRNA and protein expression in the liver tissues of Tibetan pigs remains to be performed. Recently, high-throughput sequencing of DNA, RNA, and proteins, has been widely used to study physiological processes and traits [9,10] in pigs. In the present study, we combined proteomic and transcriptomic analyses to characterize liver function in Tibetan and Yorkshire pigs, to better understand the high-altitude adaptations of Tibetan pigs.

## MATERIALS AND METHODS

### Ethics statement and sample collection

The procedures for animal care were approved by the Animal Welfare Committee of the Tibet Agriculture and Animal Husbandry College, and all experiments were conducted in accordance with approved relevant guidelines and regulations. Tibetan and Yorkshire pigs were raised under identical conditions with same diet at the farm of the Tibet Agriculture and Animal Husbandry College, located in Linzhi city (Tibet, 2,900 m, above mean sea level). All pigs were housed in standard conditions with natural, uncontrolled room temperature and light. Complete formula meal feed was fed three times per day and pigs had *ad libitum* access to water. At the age of 6 months, three Tibetan pigs and three Yorkshire pigs were randomly selected to slaughter and sample. Liver tissues from each individual were collected, immediately frozen in liquid nitrogen, and stored at −80°C until use.

### RNA isolation and library construction

The total RNA from liver tissues was extracted following the phenol-chloroform method. RNA concentration and integrity were determined using an Agilent 2100 Bioanalyzer and the Agilent RNA 6000 Nano Kit (Agilent Technologies, Palo Alto, CA, USA). RNA-seq libraries were constructed using the NEBNext Ultra RNA Library Prep Kit for Illumina (# E7530L, NEB; San Diego, CA, USA) according to the manufacturer’s instructions. Paired-end 150 bp reads were generated using the HiSeq Xten platform.

### Data analysis of RNA-Seq

Data analysis included quality control of raw reads, filtering, alignment, assembly, expression count, and annotation. Contaminated reads were filtered using a Perl script in-house. Clean reads were mapped to the reference genome (*Sus scrofa* genome v11.1, downloaded from ENSEMBL web server) using HISAT2 v2.1.0 [11]. Reads for each gene in each sample were counted by HTSeq v0.6.0 (https://github.com/simon-anders/htseq). Fragments per kilobase per million mapped reads were then calculated to estimate the expression level of each gene. Deseq (http://bioconductor.org/packages/release/bioc/html/DESeq.html) was used to identify the differentially expressed genes (DEGs), with the criteria of fold change >2 and adjusted p-value <0.05 [12].

### Expression level validation by quantitative-polymerase chain reaction

Differentially expressed mRNAs were selected for validation by quantitative-polymerase chain reaction (qPCR). Total RNA was extracted from liver tissue using TRIzol reagent, according to the manufacturer’s instructions (Thermo Fisher Scientific, Waltham, MA, USA). The cDNA synthesis was performed with 1 μg of total RNA, following the protocol accompanying the FastQuant RT Kit (Tiangen Biotech (Beijing) Co., LTD, Beijing, China). The qPCR amplification was performed as described previously [1]. Relative expression is presented as mean±standard error of the mean. Differences were tested for statistical significance using the Student’s t-test. A p-value <0.05 was considered as the threshold for statistical significance (* p<0.05; ** p<0.01).

### Protein extraction and labeling

Liver tissue protein was extracted using a mammalian tissue total protein extraction kit (AP0601-50, Beijing Bangfei Biotechnology Co., Ltd., Beijing, China). Quantification was investigated using a protein quantification kit (Dingguo Changsheng, Beijing, China). Protein peptides from each group were labeled using the 8-plex isobaric tags for relative and absolute quantitation (iTRAQ) reagents multiplex kit (ABI, Foster City, CA, USA). For each sample, approximately 200 μg protein was precipitated with 10 volumes of acetone at −20°C overnight. After centrifugation, the protein pellet was dissolved in 60 μL of dissolution buffer. The iTRAQ labeling reagents were added to the peptide samples and reacted at room temperature for 1 h, before investigation of the labeling and extraction efficiency. Samples were pooled, vacuum-dried, and dissolved in solution. The reconstituted peptides were analyzed with a Q-Exactive mass spectrometer (Thermo Fisher Scientific, USA), coupled with a nano high-performance liquid chromatography system (Thermo Fisher Scientific, USA). The detailed steps for enzymolysis and iTRAQ labeling have been described previously [5,9].

### Retrieval of database and protein data analysis

Data analysis was performed using Proteome Discover v2.1 (Thermo Fisher Scientific, USA) software. Peptide identification was performed using the SEQUEST search engine, using the Uniprot database (*Sus scrofa* 49003 entries, 20190102). Proteins were identified according to the following criteria: mass error was set to 15 ppm for precursor ions and 0.02 Da for fragment ions. Trypsin was used as a cleavage enzyme, and two missing cleavages were allowed. The false discovery rate was adjusted to 0.01. Differentially expressed proteins (DEPs) were identified as having a fold change >1.2 and p-value <0.05.

### Functional enrichment analysis

Gene ontology (GO) and Kyoto encyclopedia of genes and genomes (KEGG) functional enrichment analyses were performed, using the g:Profiler web server [13]. Visualization of enrichment results was performed using the R packages ‘ggplot2’, ‘forcats’, ‘enrichplot’, ‘wordcloud2’, and ‘GOplot’.

## RESULTS AND DISCUSSION

### Transcriptomic profiles in RNA-Seq

**Figure 1:**
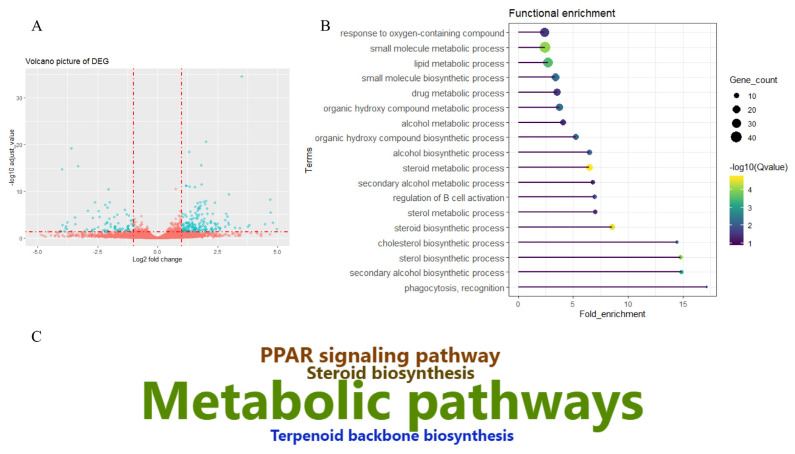
Differentially expressed genes and functional enrichment analysis. (A) Volcano plot of differentially expressed genes (DEGs) between Tibetan and Yorkshire pigs. (B) Relevant biological processes enriched by DEGs. (C) Kyoto encyclopedia of genes and genomes pathway enriched by DEGs.

After removing low quality reads, an average of 45.86 million paired-end reads were obtained for each sample. The mapping rate aligning to *Sus scrofa* 11.1 of all samples was above 96.2%. Reads located on exons accounted for more than 68.71% (Supplementary Table S1). A total of 18,791 genes were identified across the six libraries. A number of genes highly expressed in liver tissue, such as albumin, fibrinogen beta chain precursor, cytochrome c oxidase subunit I, apolipoprotein E precursor, catalase, cytochrome P450 2E1, ATP synthase F0 subunit 6, vitronectin, apolipoprotein A-I (*APOA1*), and 17-beta-hydroxysteroid dehydrogenase 13, may play important roles in determining the metabolic pattern of liver tissue. Tibetan pigs have adapted to a high-altitude environment, and their livers have adapted to function under hypoxia and low temperatures. The current study aimed to characterize the molecular adaptation pattern by comparing the DEGs between Tibetan and Yorkshire (low-land breed) pigs; after a series of filtering steps, 273 DEGs were identified, which included 187 upregulated and 86 downregulated genes in the Tibetan group relative to the Yorkshire group (Figure 1A; Supplementary Table S2).

### Functional enrichment analysis of differentially expressed genes

To further understand the potential molecular mechanisms involved in high-altitude adaptation, DEGs were subjected to functional enrichment analysis (Supplementary Table S3). Relevant GO classifications were identified (Figure 1B). These included the response to oxygen-containing compounds, the small molecule biosynthetic or metabolic process, the lipid metabolic process, the drug metabolic process, the organic hydroxyl compound metabolic process, the steroid biosynthetic or metabolic process, and the cholesterol biosynthetic process. Only four KEGG pathways were enriched, which consisted of metabolic pathways, the peroxisome proliferator-activated receptor signaling pathway, steroid biosynthesis, and terpenoid backbone biosynthesis (Figure 1C; Supplementary Table S3).

### Proteomic profiles in isobaric tags for relative and absolute quantitation

A total of 19,622 peptides and 3,781 proteins were identified, of which 3,390 proteins were quantified. Protein molecular weights ranged from 20 kDa to 50 kDa. Peptide coverage, peptide count, and isoelectric points for these proteins are presented in Supplementary Figure S1. To better understand the function of these expressed protein, proteins were subjected to GO enrichment analysis. The top 10 enriched GO classifications are shown in Supplementary Figure S2, grouped according to the major GO categories of biological process, cellular component, and molecular function. For the biological process category, most proteins participated in the metabolic process (GO:0008152), the organic substance metabolic process (GO:0071704), the cellular metabolic process (GO:0044237), and the primary metabolic process (GO:0044238). For the molecular function category, most proteins were involved in binding (GO:0005488), catalytic activity (GO: 0003824), protein binding (GO:0005515), ion binding (0043167), and organic cyclic compound binding (GO:0097159). Finally, for the cellular component category, most proteins were found in the cell (GO:0005623), the cell parts (GO:0044464), the intracellular parts (GO:0005622), the cytoplasm (GO:0005737), and in intracellular organelles (GO:0043229) (Supplementary Figure S2).

### Identification of differentially expressed proteins

**Figure 2:**
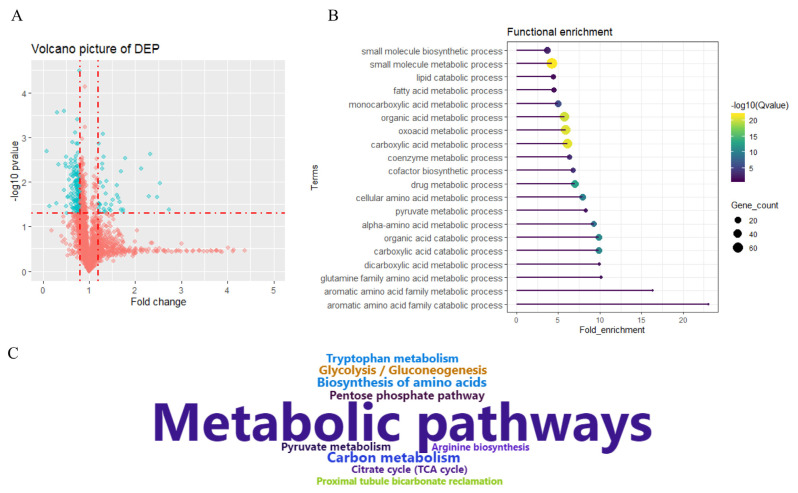
Differentially expressed proteins and functional enrichment analysis. (A) Volcano plot of differentially expressed proteins (DEPs) between Tibetan and Yorkshire pigs. (B) Relevant biological processes enriched by DEPs. (C) Kyoto encyclopedia of genes and genomes pathway enriched by DEPs.

Using 1.20/0.833 and a p-value <0.05 as a threshold to classify proteins as upregulated or downregulated, a total of 257 DEPs were identified in Tibetan pigs relative to Yorkshire pigs. These proteins consisted of 38 upregulated proteins and 219 downregulated proteins (Figure 2A; Supplementary Table S4). The most highly expressed proteins in the Tibetan group were phosphatidylinositol-4-phosphate 3-kinase catalytic subunit type 2 gamma (PIK3C2G), myoglobin (MB), carbonyl reductase (CBR2), solute carrier family 9 member C1 (SLC9C1), PDZ and LIM domain 5 (PDLIM5), MYL3, LOC100736765 (Myosin-7), troponin C (TNNC1), and TNNT2 (Supplementary Table S4). The most highly expressed proteins in the Yorkshire group were ADH4, CAD, LOC100739663 (metallothionein), metallothionein-1D (MT1D), keratin 75 (KRT75), LOC110260333 (3-hydroxy-3-methylglutaryl coenzyme A synthase), keratin 5 (KRT5), and fatty acid synthase (FASN) (Supplementary Table S4).

### Functional enrichment analysis of differentially expressed proteins

To further characterize protein expression patterns, DEPs were subjected to functional enrichment analysis. Relevant terms associated with GO biological processes and KEGG pathways are presented in Figures 2B and 2C, respectively, and the detailed information is presented in Supplementary Table S5. Relevant terms were mainly divided into metabolic processes, biosynthetic processes, and catabolic processes. The biosynthetic processes consist of small molecule biosynthetic and cofactor biosynthetic processes. These metabolic processes included carboxylic acid, the aromatic amino acid family, fatty acid metabolic, monocarboxylic acid, organic, oxoacid, carboxylic, coenzyme, drug, cellular amino acid, pyruvate, alpha-amino acid, dicarboxylic acid, and glutamine family amino acid processes. The catabolic processes included the carboxylic acid catabolic process, aromatic amino acid family catabolic process, lipid, organic acid, carboxylic acid, and aromatic amino acid processes (Figure 2B). KEGG pathways enriched by DEPs consisted of metabolic pathways, carbon metabolism, glycolysis/gluconeogenesis, biosynthesis of amino acids, the pentose phosphate pathway, tryptophan metabolism, pyruvate metabolism, arginine biosynthesis, the citrate cycle, and proximal tubule bicarbonate reclamation (Figure 2C).

### Real-time quantitative polymerase chain reaction verification

**Figure 3:**
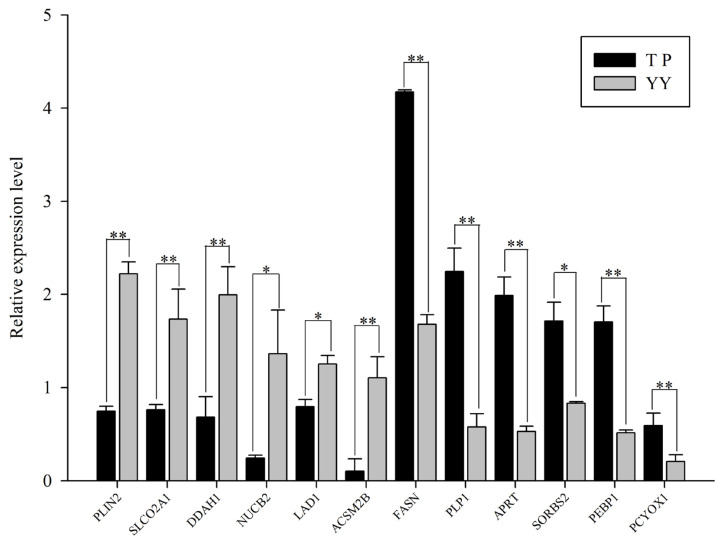
Validation of differentially expressed genes with quantitative polymerase chain reaction. * p<0.05; ** p<0.01.

To validate the RNA-Seq results, 12 DEGs were randomly selected for qPCR analysis (Figure 3). Of these, acyl-CoA synthetase medium chain family member 2B (*ACSM2B*), dimethylarginine dimethylaminohydrolase 1 (*DDAH1*), adenine phosphoribosyltransferase (*APRT*), phosphatidylethanolamine binding protein 1, *FASN*, proteolipid protein 1, sorbin and SH3 domain containing 2 (*SORBS2*), and nucleobindin 2 were downregulated, and solute carrier organic anion transporter family member 2A1 (*SLCO2A1*), prenylcysteine oxidase 1 (*PCYOX1*), perilipin 2 (*PLIN2*), and ladinin 1 (*LAD1*) were upregulated. Figure 3 shows the relative expression levels between the two groups, which are consistent with the RNA-Seq data, indicating that identification and abundance estimates of genes are highly reliable in this study.

### Integrated analysis of DEGs, DEPs, and published data

**Figure 4:**
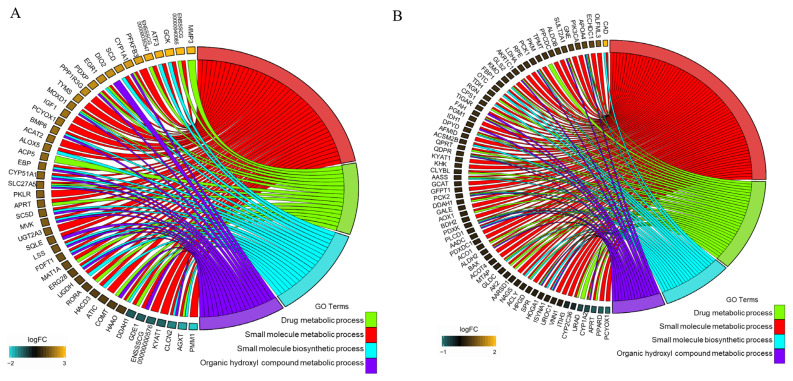
Overlapping functional enrichment result between DEGs and DEPs. The curved lines connect gene ontology categories with their associated DEGs (A) or DEPs (B). The color shading is proportional to the fold change values of the differential expression level, which were obtained from Tibetan vs Yorkshire pigs, and then transformed into logFC values (log_2_ fold change). DEGs, differentially expressed genes; DEPs, differentially expressed proteins.

Cumulatively, 18,791 genes and 3,390 proteins were investigated and compared; 273 DEGs and 257 DEPs, of which 216 proteins were annotated, were identified. Only 11 genes overlapped between the DEGs and DEPs (Table 1). Of these, nine DEGs showed the same expression pattern. The five upregulated genes at both mRNA and protein levels included retinol dehydrogenase 16 (*RDH16*), *APRT*, *PCYOX1*, *SORBS2*, and *ENSSSCG00000036224* (Table 1). RDH16, retinol dehydrogenase 16, has been shown to be reduced by insulin in HepG2 cells, and to regulate energy balance and adiposity [14]. PCYOX1 hydrolyzes the thioether bond of prenylcysteines in the final step of degradation of prenylated proteins and propagates the oxidative burden of low-density lipoproteins [15]. SORBS2, sorbin and SH3 domain-containing 2, could suppress hepatocellular carcinoma tumorigenesis and metastasis [16]. Four downregulated genes at both mRNA and protein levels included *PLIN2*, *LAD1*, kynurenine aminotransferase 1 (*KYAT1*), and *DDAH1* (Table 1). PLIN2 has been shown to induce obesity and progressive fatty liver disease via mechanistically distinct hepatocyte and extra-hepatocyte actions [17]. KYAT1 plays a role in protecting against liver damage from thioacetamide [18]. Dimethylarginine dimethylaminohydrolase 1 functions in protecting against hepatic steatosis induced by a high-fat diet and insulin resistance [19]. In addition, four GO enrichment terms overlapped those from the DEGs (Figure 4A) and those from the DEPs (Figure 4B). These four classifications included the small molecule metabolic process, the small molecule biosynthetic process, the drug metabolic process, and the organic hydroxyl compound metabolic process (Figure 4). This shows that biosynthetic and metabolic patterns of small molecular and organic hydroxyl compound metabolic processes were functional in the high-altitude adapted group. Genes involved in these biological processes are presented in Figure 4. Oxygen content is less in a high-altitude environment. Populations in this region have undergone natural selection, leading to improved oxygen delivery against the challenging environmental stress [20]. Growing evidence also suggests that high-altitude adaptation is derived from multiple molecular mechanisms; not only oxygen delivery but also oxygen utilization by cellular metabolism [21,22]. It was reported that decreased fatty acid oxidation could also be a better strategy of hypoxia adaptation since oxidation of fatty acids generates less ATP than carbohydrates per molecule of oxygen consumed [23]. In this study, proteins involved in the fatty acid metabolic process (including 15-hydroxyprostaglandin dehydrogenase, ATP citrate lyase [*ACLY*], acyl-CoA thioesterase 4, 3-hydroxybutyrate dehydrogenase 2, phosphoenolpyruvate carboxykinase 2 [*PCK2*], *ACSM2B*, aldo-keto reductase family 1 member C1, *PCK1*, apolipoprotein A4 and ethylmalonyl-CoA decarboxylase 1) were lower in Tibetan pigs compared to the Yorkshire pigs. Another change induced by hypoxia could be the conversion of glucose oxidation to glycolysis to decrease oxygen demand and maintain energy production [24,25]. This condition would promote glucose uptake and glycolysis and suppress mitochondrial glucose oxidation. Oxygen demand and utilization was suppressed in the hypoxia adaptation process through a decreasing mitochondrial density and the expression level of tricarboxylic acid cycle (TCA) enzymes [26]. In this study, protein level of five enzymes of the TCA cycle pathway (isocitrate dehydrogenase 1, *ACLY*, aconitase 1, *PCK2*, and *PCK1*) were lower in the Tibetan pig relative to the Yorkshire pig (a low-land breed)(Supplementary Table S5, Table S4). Furthermore, genes involved in glycolysis (glucokinase and phosphomannomutase 1) were upregulated in the Tibetan group relative to the Yorkshire group. Conversely, genes related to the gluconeogenesis process (*PCK1*, *PCK2*, and fructose-bisphosphatase 1) were decreased in the Tibetan group. This may imply that glycolysis is more active in the Tibetan pig to reduce oxygen demand and maintain energy production. In this case, it could be deduced that oxygen utilization efficiency was higher in the Tibetan pig than in the low-land pig after long-term, high-altitude adaptation, which mainly depended on reducing fatty acid metabolism and glucose oxidation, and increasing glycolysis. Undoubtedly, these differences between Tibetan and Yorkshire pigs maybe also derived from their different genetic backgrounds. The experimental animals in this study were raised in a high-land region. If the data from the counterparts of the Tibetan and Yorkshire pigs raised in the low altitude area were integrated in this study, key genes could be recognized precisely in the different environments and breeds.

Results of a similar study [2] on the molecular characterization of liver mRNA between Tibetan and Rongchang pigs were compared as in the present study. They compared gene expression levels using RNA-Seq between Tibetan pigs (high-land model) and Rongchang pigs (low-land model) and identified 490 DEGs. Compared to the present study, 22 DEGs and 10 DEPs were found to overlap (Table 1). Of these 22 DEGs, nine DEGs (*ENSSSCG00000006985*, nocturnin, *SLCO2A1*, squalene epoxidase [*SQLE*], UDP-glucose 6-dehydrogenase, Rho guanine nucleotide exchange factor 16, klotho beta, nei like DNA glycosylase 1, and cortactin binding protein 2) showed a similar expression pattern in both studies. The remaining 13 genes (Fos proto-oncogene, growth arrest and DNA damage inducible beta, insulin like growth factor 1, *SQLE*, FAD dependent oxidoreductase domain containing 2, TNF alpha induced protein 3, early growth response 1, solute carrier family 1 member 1, *ENSSSCG 00000016119*, *ENSSSCG00000006716*, RAR related orphan receptor A, 6-phosphofructo-2-kinase/fructose-2,6-biphosphatase 3, neural EGFL like 2, and Ras association domain family member 6) showed the opposite pattern between studies. Furthermore, 10 DEPs were found to overlap with this previous study. Four proteins (NADH:ubiquinone oxidoreductase subunit B5, aminoadipate-semialdehyde synthase, collagen type XIV alpha 1 chain, and nerve growth factor receptor) showed a similar expression pattern, while six (phytanoyl-CoA dioxygenase domain containing 1, CXXC motif containing zinc binding protein, nudix hydrolase 6, thiopurine S-methyltransferase, decapping enzyme, scavenger, and UDP-galactose-4-epimerase) showed the opposite expression. The divergence between groups could be explained by different living environments and molecular genetic characterization.

## IMPLICATIONS

Herein, 273 and 257 differentially expressed genes and proteins were identified in Tibetan and Yorkshire pigs, respectively. Evidence from functional enrichment analysis showed many genes were involved in metabolic processes. The combined functional enrichment result from differentially expressed genes and proteins revealed that small molecular biosynthesis, metabolic and organic hydroxyl compound metabolic processes, were predominantly different between the two breeds. To reduce oxygen consumption and maintain energy production, glycolysis may be preferred to fatty acid metabolism and glucose oxidation in Tibetan pigs for adaptation to hypoxia. These findings provide novel insight into the high-altitude metabolic adaptations of Tibetan pigs.

## Figures and Tables

**Table 1:** Comparison of differentially expressed genes between pairwise groups

Group	Gene_id	Gene name or description	Fold change		

			DEG^1)^	DEP^2)^	Reported^1)^
Reported_DEG_vs_DEG	ENSSSCG00000006985	*-*	−3.8	-	−1.40
Reported_DEG_vs_DEG	ENSSSCG00000024596	*NOCT*	−1.8	-	−2.77
Reported_DEG_vs_DEG	ENSSSCG00000011641	*SLCO2A1*	−2.4	-	−1.95
Reported_DEG_vs_DEG	ENSSSCG00000002383	*FOS*	3.5	-	−2.64
Reported_DEG_vs_DEG	ENSSSCG00000022689	*GADD45B*	2.1	-	−1.45
Reported_DEG_vs_DEG	ENSSSCG00000000857	*IGF1*	1.9	-	−2.69
Reported_DEG_vs_DEG	ENSSSCG00000005970	*SQLE*	1.5	-	1.13
Reported_DEG_vs_DEG	ENSSSCG00000000142	*FOXRED2*	1.0	-	−1.36
Reported_DEG_vs_DEG	ENSSSCG00000030150	*UGDH*	1.2	-	1.44
Reported_DEG_vs_DEG	ENSSSCG00000004154	*TNFAIP3*	1.7	-	−1.00
Reported_DEG_vs_DEG	ENSSSCG00000014336	*EGR1*	2.3	-	−1.03
Reported_DEG_vs_DEG	ENSSSCG00000005222	*SLC1A1*	−1.8	-	0.83
Reported_DEG_vs_DEG	ENSSSCG00000024223	*ARHGEF16*	−3.6	-	−1.79
Reported_DEG_vs_DEG	ENSSSCG00000026297	*KLB*	1.3	-	1.46
Reported_DEG_vs_DEG	ENSSSCG00000016119	*-*	1.2	-	−0.80
Reported_DEG_vs_DEG	ENSSSCG00000001882	*NEIL1*	1.1	-	0.94
Reported_DEG_vs_DEG	ENSSSCG00000006716	*-*	−2.1	-	5.49
Reported_DEG_vs_DEG	ENSSSCG00000004576	*RORA*	1.1	-	−1.19
Reported_DEG_vs_DEG	ENSSSCG00000016625	*CTTNBP2*	−1.2	-	−0.95
Reported_DEG_vs_DEG	ENSSSCG00000011133	*PFKFB3*	2.6	-	−0.98
Reported_DEG_vs_DEG	ENSSSCG00000000802	*NELL2*	1.6	-	−1.03
Reported_DEG_vs_DEG	ENSSSCG00000008952	*RASSF6*	1.3	-	−2.53
DEG_vs_DEP	ENSSSCG00000000419	*RDH16*	2.0	2.32	-
DEG_vs_DEP	ENSSSCG00000002650	*APRT*	1.6	1.60	-
DEG_vs_DEP	ENSSSCG00000029790	*PCYOX1*	1.9	1.74	-
DEG_vs_DEP	ENSSSCG00000009851	*PEBP1*	1.2	0.83	-
DEG_vs_DEP	ENSSSCG00000035863	*PLIN2*	−2.2	0.71	-
DEG_vs_DEP	ENSSSCG00000036679	*SORBS2*	1.0	1.28	-
DEG_vs_DEP	ENSSSCG00000021624	*LAD1*	−2.0	0.77	-
DEG_vs_DEP	ENSSSCG00000005663	*KYAT1*	−1.2	0.75	-
DEG_vs_DEP	ENSSSCG00000036224	*-*	2.6	1.34	-
DEG_vs_DEP	ENSSSCG00000040735	*DDAH1*	−1.2	0.78	-
DEG_vs_DEP	ENSSSCG00000029944	*FASN*	1.8	0.46	-
Reported_DEG_vs_DEP	ENSSSCG00000011764	*NDUFB5*	-	1.67	0.90
Reported_DEG_vs_DEP	ENSSSCG00000005665	*PHYHD1*	-	0.67	0.89
Reported_DEG_vs_DEP	ENSSSCG00000016613	*AASS*	-	0.77	−1.77
Reported_DEG_vs_DEP	ENSSSCG00000003850	*CZIB*	-	0.77	1.10
Reported_DEG_vs_DEP	ENSSSCG00000009085	*NUDT6*	-	0.82	0.77
Reported_DEG_vs_DEP	ENSSSCG00000001073	*TPMT*	-	0.71	0.90
Reported_DEG_vs_DEP	ENSSSCG00000015231	*DCPS*	-	0.73	1.30
Reported_DEG_vs_DEP	ENSSSCG00000005997	*COL14A1*	-	0.71	−1.07
Reported_DEG_vs_DEP	ENSSSCG00000027827	*GALE*	-	0.78	0.83
Reported_DEG_vs_DEP	ENSSSCG00000017548	*NGFR*	-	0.65	−2.09

*NOCT*, nocturnin; *SLCO2A1*, solute carrier organic anion transporter family member 2A1; *FOS*, Fos proto-oncogene; *GADD45B*, growth arrest and DNA damage inducible beta; *IGF1*, insulin like growth factor 1; *SQLE*, squalene epoxidase; *FOXRED2*, FAD dependent oxidoreductase domain containing 2; *UGDH*, UDP-glucose 6-dehydrogenase; *TNFAIP3*, TNF alpha induced protein 3; *EGR1*, early growth response 1; *SLC1A1*, solute carrier family 1 member 1; *ARHGEF16*, Rho guanine nucleotide exchange factor 16; *KLB*, klotho beta; *NEIL1*, nei like DNA glycosylase 1; *RORA*, RAR related orphan receptor A; *CTTNBP2*, cortactin binding protein 2; *PFKFB3*, 6-phosphofructo-2-kinase/fructose-2,6-biphosphatase 3; *NELL2*, neural EGFL like 2; *RASSF6*, Ras association domain family member 6; *RDH16*, retinol dehydrogenase 16; *APRT*, adenine phosphoribosyltransferase; *PCYOX1*, prenylcysteine oxidase 1; *PEBP1*, phosphatidylethanolamine binding protein 1; *PLIN2*, perilipin 2; *SORBS2*, sorbin and SH3 domain containing 2; *LAD1*, ladinin 1; *KYAT1*, kynurenine aminotransferase 1; *DDAH1*, dimethylarginine dimethylaminohydrolase 1; *FASN*, fatty acid synthase; *NDUFB5*, NADH:ubiquinone oxidoreductase subunit B5; *PHYHD1*, phytanoyl-CoA dioxygenase domain containing 1; *AASS*, aminoadipate-semialdehyde synthase; *CZIB*, CXXC motif containing zinc binding protein; *NUDT6*, nudix hydrolase 6; *TPMT*, thiopurine S-methyltransferase; *DCPS*, decapping enzyme, scavenger; *COL14A1*, collagen type XIV alpha 1 chain; *GALE*, UDP-galactose-4-epimerase; *NGFR*, nerve growth factor receptor; DEG, differentially expressed genes; DEP, differentially expressed protein.

1) −log_2_(fold change).

2) Fold change.
