# Supplementary material for: Integrated analysis of transcriptomic and proteomic analyses reveals different metabolic patterns in the livers of Tibetan and Yorkshire pigs
Source: Anim Biosci. 2020 Oct 13;34(5):922–30. doi: 10.5713/ajas.20.0342 (PMC8100475; doi:10.5713/ajas.20.0342)
Supplement: Supplementary file 1 [file ajas-20-0342-suppl.pdf]

SUPPORTING INFORMATION

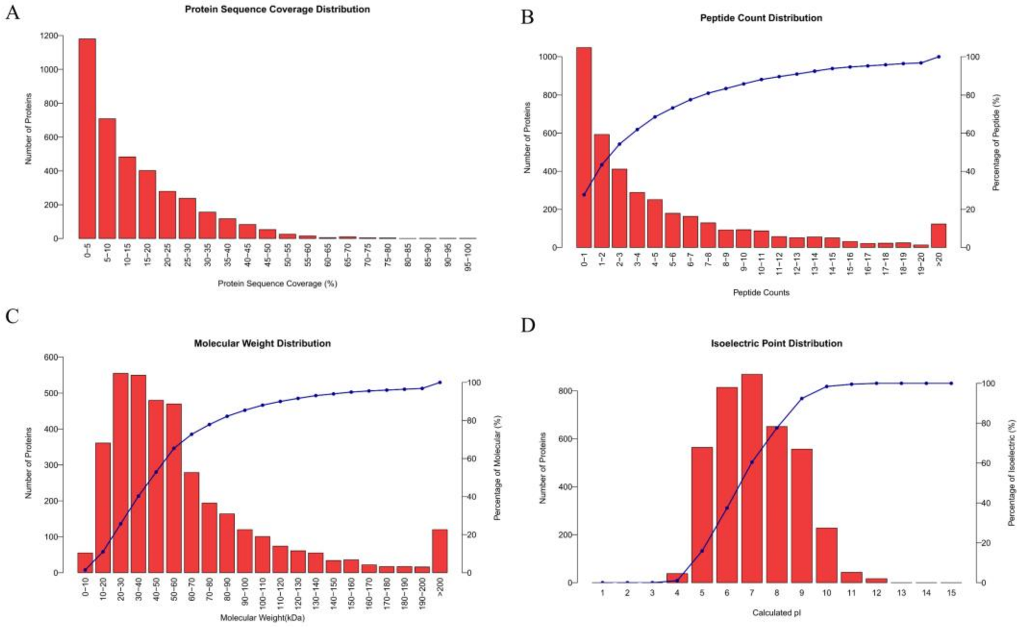

Supplementary Figure S1. Overview of protein identification information. (A) Distribution of protein coverage by the peptides identified. (B) Distribution of proteins containing different numbers of identified peptides. (C) Distribution of the proteins identified according to molecular weight. (D) Distribution of the proteins identified according to isoelectric point.

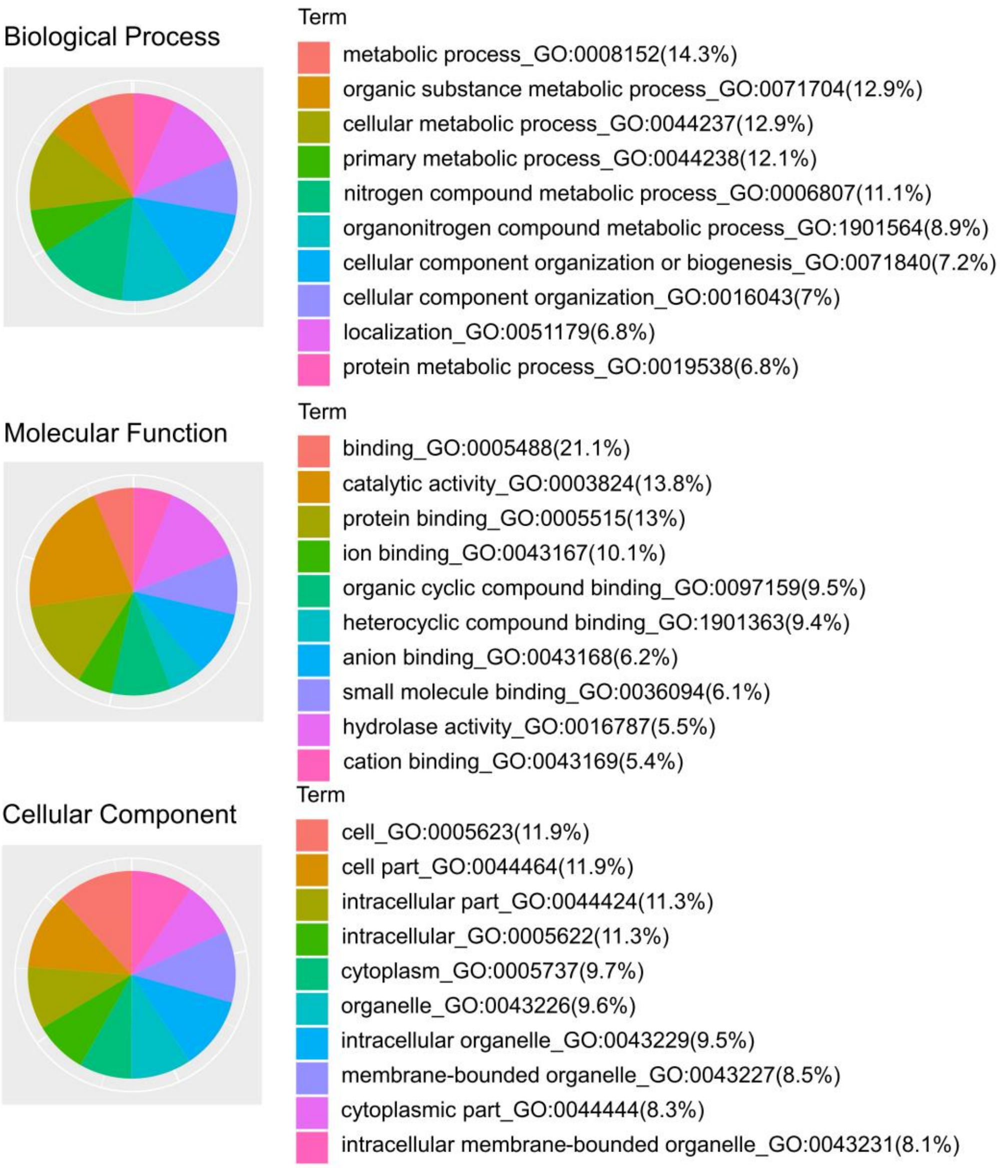

Supplementary Figure S2. Functional enrichment analyses for identified proteins. Biological process (upper); molecular function (middle); cellular component (bottom).

Table S1. Summary of RNA-Seq reads and alignment.

Table S2. List of differentially expressed genes between the Tibetan and Yorkshire groups.

Table S3. Result of functional enrichment analysis enriched by differentially expressed genes between the Tibetan and Yorkshire groups.

Table S4. List of differentially expressed proteins between the Tibetan and Yorkshire groups.

Table S5. Result of functional enrichment analysis enriched by differentially expressed proteins between the Tibetan and Yorkshire groups.

Table S1 mapping rate

Group	Sample	Total Reads	Mapped Reads	Mapping Rate	UnMapped Reads	MultiMap Reads	MultiMap Rate	Exon (rate)	Intron(rate)	Intergenic(rate)
TP	TP1	44,386,672	42,745,703	0.963	1,640,969	1,751,215	0.0395	11,552,188(68.71%)	3,635,090(21.62%)	1,624,598(9.66%)
TP	TP2	47,641,488	45,887,425	0.9632	1,754,063	1,781,882	0.0374	12,369,531(68.91%)	3,910,694(21.79%)	1,670,731(9.31%)
TP	TP3	46,469,190	44,716,490	0.9623	1,752,700	1,940,313	0.0418	12,127,356(69.13%)	3,786,020(21.58%)	1,629,974(9.29%)
YP	YY1	47,877,574	46,356,804	0.9682	1,520,770	1,643,504	0.0343	13,726,671(70.62%)	4,205,612(21.64%)	1,505,270(7.74%)
YP	YY2	44,436,712	42,816,334	0.9635	1,620,378	1,480,293	0.0333	12,426,279(69.76%)	3,707,587(20.81%)	1,678,240(9.42%)
YP	YY3	44,339,406	42,927,709	0.9682	1,411,697	1,311,670	0.0296	13,834,453(72.07%)	3,689,502(19.22%)	1,673,019(8.72%)

Table S2 DEG list

Gene	TP1_count	TP2_count	TP3_count	YY1_count	YY2_count	YY3_count	FoldChange	Log2FoldChange	pval	padj	Up/Down	GeneName
ENSSSCG00000000061	323	211	396	1619	1069	3453	0.182051837	-2.457578799	5.83E-09	2.03E-06	down	PMM1
ENSSSCG00000000142	118	136	96	64	62	66	2.069385838	1.049202661	0.00017608	0.010322706	up	FOXRED2
ENSSSCG00000000399	201	200	191	71	76	53	3.305323859	1.724791635	4.48E-08	1.20E-05	up	TIMELESS
ENSSSCG00000000419	15268	12871	16922	4616	5406	2728	3.876951444	1.954922666	4.32E-07	8.27E-05	up	RDH16
ENSSSCG00000000475	64	160	92	37	44	42	2.885669008	1.528905829	0.000430943	0.020142202	up	IRAK3
ENSSSCG00000000576	2877	3516	5597	12590	9647	9224	0.423984793	-1.237915574	0.00033362	0.016994653	down	ENSSSCG00000000576
ENSSSCG00000000591	717	839	393	365	281	305	2.309692715	1.207700926	0.000939639	0.035303939	up	PIK3C2G
ENSSSCG00000000802	313	719	445	165	231	127	3.092005167	1.62854273	0.000229975	0.012714981	up	NELL2
ENSSSCG00000000857	194	231	266	103	65	31	3.754501465	1.908621355	0.000308673	0.015974932	up	IGF1
ENSSSCG00000000901	559	302	491	198	232	236	2.320086283	1.214178459	0.000168676	0.010069535	up	FGD6
ENSSSCG00000000997	135	158	531	77	66	40	4.912550603	2.296472268	0.00033125	0.01693736	up	PPP1R3G
ENSSSCG00000001027	720	810	983	300	275	187	3.638991619	1.863538729	3.20E-08	9.27E-06	up	BMP6
ENSSSCG00000001441	66	15	16	0	2	0	54.8507781	5.777440181	5.77E-05	0.004432993	up	ENSSSCG00000001441
ENSSSCG00000001565	247	444	633	207	190	146	2.689220151	1.427187865	0.000783469	0.031095144	up	CDKN1A
ENSSSCG00000001683	628	606	618	272	114	353	2.937861794	1.554766529	3.22E-05	0.002754338	up	POLH
ENSSSCG00000001723	107	182	220	767	728	790	0.250653873	-1.996231568	3.93E-11	2.47E-08	down	PLA2G7
ENSSSCG00000001844	31	35	45	172	112	87	0.329435736	-1.601931031	0.000457195	0.02100778	down	PLIN1
ENSSSCG00000001867	139	117	114	58	64	48	2.445450437	1.290100225	9.02E-05	0.00632661	up	PSTPIP1
ENSSSCG00000001882	1666	1525	1084	721	809	801	2.083098855	1.058731305	3.60E-05	0.003039529	up	NEIL1
ENSSSCG00000001906	1380	9149	4255	1006	1101	674	5.760091472	2.526091722	6.55E-05	0.004789982	up	CYP1A1
ENSSSCG00000001978	46	61	59	24	22	25	2.656842028	1.40971245	4.82E-05	0.003876207	up	GZMB
ENSSSCG00000002313	5188	6116	5223	2592	2209	1116	3.037340692	1.602808741	0.000141613	0.008917012	up	SLC10A1
ENSSSCG00000002383	8489	15857	10424	1132	944	1415	11.37769608	3.508136544	2.30E-39	3.12E-35	up	FOS
ENSSSCG00000002436	374	400	490	155	194	286	2.308744707	1.207108654	1.05E-07	2.49E-05	up	TTC7B
ENSSSCG00000002650	874	588	835	266	243	388	2.976269365	1.573505102	4.00E-10	1.94E-07	up	APRT
ENSSSCG00000002825	22011	16477	12957	6501	7067	8369	2.696527107	1.431102537	6.50E-08	1.67E-05	up	ENSSSCG00000002825
ENSSSCG00000002847	6745	3474	2379	12570	17918	19606	0.291883026	-1.776537778	4.90E-05	0.003898105	down	GPT2
ENSSSCG00000003044	47	47	48	15	27	15	2.789543575	1.480029088	0.00130874	0.044169166	up	LYPD4
ENSSSCG00000003140	122	59	355	54	39	42	4.451893132	2.154418961	0.001453003	0.047505522	up	FGF21
ENSSSCG00000003142	107	64	75	28	38	48	2.50372921	1.324078537	0.000501096	0.022345588	up	IZUMO1
ENSSSCG00000003148	103	232	272	90	47	113	2.796838748	1.483797078	0.001362485	0.044978528	up	DBP
ENSSSCG00000003155	723	591	740	173	178	353	3.440998947	1.782827451	3.99E-11	2.47E-08	up	PPP1R15A
ENSSSCG00000003551	763	574	524	239	294	150	3.008315495	1.588955877	0.000101774	0.007026507	up	PAQR7
ENSSSCG00000003882	668	440	434	170	226	331	2.485542964	1.313561041	2.37E-05	0.002148361	up	SLC5A9
ENSSSCG00000003891	14272	13802	9678	6033	4174	8254	2.383689857	1.253196538	4.64E-06	0.000630915	up	CYP4A24
ENSSSCG00000003892	79	59	235	5	1	11	26.26168863	4.71488778	1.04E-08	3.45E-06	up	ENSSSCG00000003892
ENSSSCG00000004049	622	458	376	144	157	180	3.475187723	1.797090911	2.69E-10	1.36E-07	up	ACAT2
ENSSSCG00000004154	443	511	728	218	183	192	3.188544363	1.672897954	4.49E-09	1.70E-06	up	TNFAIP3
ENSSSCG00000004157	0	4	4	32	25	41	0.093336141	-3.421420369	1.19E-05	0.001252421	down	IL20RA
ENSSSCG00000004191	297	521	764	178	165	118	3.774014233	1.916099864	1.17E-05	0.001249333	up	MOXD1
ENSSSCG00000004419	194	196	212	305	366	775	0.494966269	-1.014597883	0.001331621	0.044306533	down	MFSD4B
ENSSSCG00000004576	865	463	625	393	302	322	2.177508954	1.122678651	0.001236634	0.042688982	up	RORA
ENSSSCG00000004628	83	108	85	30	46	36	2.781492349	1.475859137	1.47E-05	0.001451428	up	TMOD2
ENSSSCG00000004891	42	79	78	13	6	25	5.324425759	2.412625939	1.05E-05	0.001148853	up	SERPINB7

ENSSSCG00000004902	82	85	84	187	213	640	0.293179824	-1.770142269	6.41E-05	0.004762514	down	RNF152
ENSSSCG00000004935	1931	1516	1081	818	780	826	2.125349441	1.087700063	0.000232291	0.012791026	up	HACD3
ENSSSCG00000005004	131	107	108	58	35	51	2.741925514	1.45518938	8.89E-06	0.001028505	up	LRR1
ENSSSCG00000005102	350	283	512	183	139	188	2.55904575	1.35560594	1.18E-05	0.001249383	up	AEN
ENSSSCG00000005222	153	488	294	947	757	2102	0.290142996	-1.785163991	0.000212365	0.012034878	down	SLC1A1
ENSSSCG00000005338	179	243	119	96	86	104	2.150944258	1.104970138	0.001208715	0.041947085	up	RECK
ENSSSCG00000005610	104	112	151	418	514	258	0.337629506	-1.566487105	0.000172475	0.01024381	down	SLC2A8
ENSSSCG00000005663	530	357	426	829	1438	1296	0.42138419	-1.246791909	6.86E-05	0.004961923	down	KYAT1
ENSSSCG00000005905	92	108	96	49	46	46	2.376347203	1.248745641	6.80E-06	0.000819019	up	SLC39A4
ENSSSCG00000005970	1392	1044	701	501	393	382	2.771711125	1.470776904	5.51E-05	0.00427643	up	SQLE
ENSSSCG00000006023	371	503	291	188	229	177	2.192992548	1.13290091	0.000499592	0.022345588	up	SYBU
ENSSSCG00000006074	241	217	254	630	426	715	0.462579686	-1.112226184	1.22E-06	0.000204746	down	STK3
ENSSSCG00000006309	708	847	680	371	297	238	2.735942035	1.452037665	3.47E-06	0.000512273	up	CD247
ENSSSCG00000006524	156	105	215	45	54	40	3.842935979	1.942208942	2.61E-06	0.000412106	up	THBS3
ENSSSCG00000006624	5099	7138	6348	3534	3301	2939	2.120946582	1.084708286	1.01E-05	0.001118268	up	SELENBP1
ENSSSCG00000006716	254	768	214	2574	1960	1102	0.236487414	-2.080164689	0.000544742	0.023823254	down	ENSSSCG00000006716
ENSSSCG00000006862	481	470	540	218	232	286	2.320234134	1.214270395	5.62E-15	6.95E-12	up	VCAM1
ENSSSCG00000006940	1758	1913	2725	605	839	829	3.194307944	1.675503401	7.03E-11	3.98E-08	up	CYR61
ENSSSCG00000006985	1	3	5	58	61	16	0.07100697	-3.815895547	4.32E-05	0.003586572	down	ENSSSCG00000006985
ENSSSCG00000007093	76	59	45	157	159	169	0.422809986	-1.241918643	7.25E-05	0.005192095	down	ZNF133
ENSSSCG00000007436	19	66	45	13	13	10	4.000749483	2.000270294	0.001329528	0.044306533	up	MMP9
ENSSSCG00000007874	133	198	191	310	431	682	0.427827561	-1.22489867	2.75E-05	0.00247333	down	ABCC1
ENSSSCG00000008013	2659	3348	2073	996	1443	629	2.875862981	1.523994941	0.000445042	0.020712948	up	IGFALS
ENSSSCG00000008217	187	215	238	94	95	76	2.701372936	1.433692824	5.41E-07	9.92E-05	up	CD8A
ENSSSCG00000008645	3187	4044	3918	1471	1607	2011	2.505660037	1.325190686	1.17E-22	3.99E-19	up	ID2
ENSSSCG00000008867	162	209	249	81	88	36	3.282568352	1.714825052	0.000380664	0.018419761	up	CTSO
ENSSSCG00000008952	1473	801	970	588	502	327	2.541796101	1.345848304	0.00151701	0.048777418	up	RASSF6
ENSSSCG00000008966	50	111	152	288	266	1206	0.218322134	-2.195469691	0.000398553	0.01902005	down	PARM1
ENSSSCG00000009181	204	239	319	74	47	142	3.429299785	1.777914029	3.95E-06	0.000558988	up	C4orf17
ENSSSCG00000009226	421	553	797	194	186	206	3.414027899	1.771474848	1.28E-09	5.27E-07	up	KLHL8
ENSSSCG00000009434	1308	2481	2130	777	673	778	2.989717109	1.580008981	1.35E-08	4.38E-06	up	RGCC
ENSSSCG00000009543	105	68	76	229	227	198	0.429321994	-1.219868011	0.000229117	0.012714981	down	MYO16
ENSSSCG00000009613	203	307	235	540	587	1955	0.294212463	-1.765069732	0.000123344	0.008026786	down	DMTN
ENSSSCG00000009673	2448	2077	2678	1197	1148	825	2.522615908	1.334920559	1.68E-05	0.00160453	up	ENSSSCG00000009673
ENSSSCG00000009827	110	128	113	255	211	347	0.498248889	-1.005061505	8.93E-07	0.000153704	down	HVCN1
ENSSSCG00000009851	10270	15369	13025	5890	6895	6810	2.22533143	1.15402022	3.84E-08	1.05E-05	up	ENSSSCG00000009851
ENSSSCG00000009931	7912	4089	3969	2259	2466	1341	2.921059701	1.546491845	0.000796246	0.031119966	up	MVK
ENSSSCG00000010008	7713	8016	5899	2124	2207	1620	4.045090219	2.01617188	1.50E-11	1.13E-08	up	SEC14L3
ENSSSCG00000010132	21272	22397	21327	9711	13349	13143	2.04163124	1.02972231	2.70E-08	7.98E-06	up	COMT
ENSSSCG00000010325	460	321	400	134	139	127	3.341606169	1.740541712	4.63E-10	2.17E-07	up	KCNMA1
ENSSSCG00000010337	16382	16213	16590	8708	8687	4113	2.492163615	1.317398787	0.001338495	0.044402139	up	MAT1A
ENSSSCG00000010394	1294	855	733	365	148	75	5.304005904	2.407082381	0.000167186	0.010061497	up	OGDHL
ENSSSCG00000010404	108	65	70	30	12	42	3.419572685	1.773816055	0.00047027	0.02132046	up	ALOX5
ENSSSCG00000010442	16	13	24	96	53	114	0.233228727	-2.100182599	3.46E-06	0.000512273	down	LIPK
ENSSSCG00000010444	21	25	12	1	1	3	13.55069748	3.760295207	4.81E-05	0.003876207	up	LIPM
ENSSSCG00000010554	1792	10529	15116	2175	1954	1402	5.400067305	2.432977389	0.000315937	0.016215336	up	SCD
ENSSSCG00000010639	4251	6229	6851	2873	2911	2646	2.297667158	1.200169823	3.81E-06	0.000545079	up	HABP2
ENSSSCG00000010728	630	2340	2420	466	347	951	3.589713174	1.843868574	0.00059002	0.025077707	up	CPXM2
ENSSSCG00000010768	2	3	2	7	10	136	0.059816066	-4.063323168	0.000978964	0.03608372	down	CFAP46
ENSSSCG00000010885	556	571	420	248	334	201	2.19303798	1.132930798	0.001208974	0.041947085	up	ENSSSCG00000010885
ENSSSCG00000010948	924	966	1070	411	445	308	2.822258526	1.496850148	3.20E-07	6.49E-05	up	CTSL
ENSSSCG00000010992	19	66	51	111	153	251	0.306412702	-1.706451989	0.000584765	0.025077707	down	ENSSSCG00000010992
ENSSSCG00000011133	193	859	456	46	46	208	6.139700384	2.618168254	0.000132238	0.008564607	up	PFKFB3
ENSSSCG00000011204	77	2	3	0	0	0	175.7740581	7.457578353	0.000490415	0.022013653	up	KCNH8
ENSSSCG00000011496	18	57	68	169	113	560	0.20680635	-2.273647612	0.000616844	0.025994493	down	ADAMTS9
ENSSSCG00000011524	766	1548	1117	475	133	374	3.963221146	1.986673471	6.36E-05	0.004759663	up	CHL1
ENSSSCG00000011561	18	26	21	71	53	58	0.401829406	-1.315344949	0.000870421	0.033161335	down	PRRT3

ENSSSCG00000011641	166	52	87	609	551	720	0.188125652	-2.410231509	1.45E-07	3.22E-05	down	SLCO2A1
ENSSSCG00000012416	67	86	60	168	122	354	0.393893096	-1.344123965	0.000623048	0.026074089	down	CHIC1
ENSSSCG00000012427	306	394	433	201	157	155	2.470596146	1.304859201	3.26E-06	0.00049884	up	ENSSSCG00000012427
ENSSSCG00000012484	200	165	150	417	341	427	0.495869304	-1.011968175	1.84E-05	0.00171156	down	SYTL4
ENSSSCG00000012521	17	29	18	57	89	143	0.259117641	-1.948320854	9.24E-06	0.001056265	down	GPRASP2
ENSSSCG00000012525	26	4	6	101	117	389	0.074197291	-3.752489675	3.16E-06	0.000488525	down	BEX1
ENSSSCG00000012536	135	127	218	8	11	1	25.92707502	4.696387552	7.90E-12	6.32E-09	up	PLP1
ENSSSCG00000012993	69	147	104	422	393	757	0.236914511	-2.077561527	7.76E-10	3.52E-07	down	SLC25A45
ENSSSCG00000013270	834	956	1044	528	314	730	2.100561886	1.07077529	0.000120481	0.0078782	up	CRY2
ENSSSCG00000013512	217	221	194	490	507	913	0.387721304	-1.366908086	2.85E-09	1.11E-06	down	PLIN4
ENSSSCG00000013513	647	556	455	1184	1257	1474	0.485095564	-1.043659107	8.31E-07	0.000146711	down	PLIN5
ENSSSCG00000013714	42	8	39	544	245	294	0.092363606	-3.436531693	1.95E-07	4.14E-05	down	ENSSSCG00000013714
ENSSSCG00000013852	56	38	38	13	15	15	3.51296644	1.812689794	5.86E-05	0.004450078	up	ENSSSCG00000013852
ENSSSCG00000013900	236	219	196	377	440	1085	0.411742414	-1.280186025	0.000510871	0.022633092	down	ENSSSCG00000013900
ENSSSCG00000014196	21	15	23	2	4	1	9.435383872	3.238081214	0.000109784	0.007319444	up	TMEM232
ENSSSCG00000014198	20	37	28	6	10	8	4.002553776	2.000920786	0.000392081	0.018777075	up	TSLP
ENSSSCG00000014336	3325	7006	3294	1298	631	1200	4.9430819	2.30541081	2.64E-08	7.97E-06	up	EGR1
ENSSSCG00000014618	366	223	288	123	168	103	2.487631923	1.314773036	0.000710671	0.028939628	up	ENSSSCG00000014618
ENSSSCG00000014875	506	510	545	183	307	193	2.549949055	1.350468424	3.50E-05	0.002977093	up	CAPN5
ENSSSCG00000014985	72	31	1	0	0	0	219.0209228	7.774924885	7.17E-05	0.005160634	up	MMP3
ENSSSCG00000015016	90	172	99	47	39	32	3.396417838	1.764013955	3.17E-05	0.002742957	up	POU2AF1
ENSSSCG00000015065	794	803	1053	246	289	320	3.533650336	1.821159289	1.02E-19	2.77E-16	up	BUD13
ENSSSCG00000015202	4	4	3	21	43	16	0.150841465	-2.728895024	0.000758479	0.030520926	down	ROBO3
ENSSSCG00000015311	3945	3551	4082	1303	1561	1106	3.251507086	1.701108568	8.97E-10	3.93E-07	up	CYP51A1
ENSSSCG00000015333	357	82	123	45	44	50	4.691914378	2.230176686	0.000303226	0.015862217	up	ASB4
ENSSSCG00000015402	1302	1856	1295	661	427	446	3.233661224	1.693168542	1.27E-07	2.92E-05	up	CACNA2D1
ENSSSCG00000015550	2212	2243	3109	866	1320	1121	2.582060114	1.368522589	5.47E-07	9.92E-05	up	RGS16
ENSSSCG00000015595	384	1547	1554	161	150	240	7.224709916	2.852939662	2.41E-08	7.44E-06	up	ATF3
ENSSSCG00000015923	455	528	410	249	282	250	2.008514848	1.006129127	1.72E-05	0.001626475	up	NOSTRIN
ENSSSCG00000016092	18	30	14	69	64	65	0.352634751	-1.50375344	0.000468175	0.021296497	down	SGO2
ENSSSCG00000016119	592	665	646	323	272	357	2.280388127	1.189279395	4.64E-15	6.91E-12	up	ENSSSCG00000016119
ENSSSCG00000016173	924	1060	1331	621	603	560	2.083399854	1.058939754	1.41E-05	0.001424327	up	ATIC
ENSSSCG00000016314	881	1806	2048	452	912	469	2.833994794	1.502837108	0.001298812	0.04394313	up	TRPM8
ENSSSCG00000016402	4324	3068	2737	24407	16950	4151	0.238150642	-2.070053657	0.001543736	0.049403199	down	AGXT
ENSSSCG00000016437	70	86	119	191	223	209	0.497408063	-1.007498202	0.00069052	0.028288432	down	WDR86
ENSSSCG00000016625	447	527	507	804	970	2286	0.436480718	-1.196010173	0.000468096	0.021296497	down	CTTNBP2
ENSSSCG00000016676	6508	3432	8067	2765	2108	1994	2.940644296	1.556132285	0.000107848	0.007297717	up	INMT
ENSSSCG00000016728	5657	4151	5640	2999	2082	1972	2.447622937	1.291381324	6.45E-05	0.004762514	up	IGFBP1
ENSSSCG00000016751	556	1102	792	21	199	19	10.79111598	3.431772165	0.000136777	0.008721319	up	GCK
ENSSSCG00000016821	170	152	165	41	71	68	3.096253549	1.630523617	3.63E-08	1.01E-05	up	SLC45A2
ENSSSCG00000016822	4307	3306	2417	1171	1007	1353	3.267751265	1.708298172	1.47E-09	5.89E-07	up	AMACR
ENSSSCG00000016850	887	1997	1532	790	675	930	2.092080057	1.06493806	0.000696035	0.028428742	up	LIFR
ENSSSCG00000016991	6453	7868	5090	2158	1803	2193	3.579618327	1.83980577	1.99E-15	3.39E-12	up	DUSP1
ENSSSCG00000017268	166	205	144	340	380	1283	0.314372863	-1.669451406	0.0004258	0.020039126	down	PRKCA
ENSSSCG00000017792	349	380	391	143	247	178	2.215317268	1.14751333	0.000155827	0.009546837	up	CORO6
ENSSSCG00000017926	183	220	93	530	596	625	0.321974735	-1.63498061	6.41E-06	0.000799233	down	SLC16A13
ENSSSCG00000017998	92	115	121	43	43	21	3.36064602	1.74873859	0.000109303	0.007319444	up	GLP2R
ENSSSCG00000018092	3111	2545	3645	7989	9075	8557	0.410397026	-1.28490782	3.30E-08	9.36E-06	down	ND6
ENSSSCG00000021418	4199	6423	4899	2013	2708	1520	2.732202229	1.450064271	5.15E-05	0.004072563	up	LSS
ENSSSCG00000021581	46	65	73	96	183	405	0.32323421	-1.629348199	0.000926593	0.035007186	down	CLCN2
ENSSSCG00000021624	267	246	129	637	858	1567	0.248952391	-2.006058221	4.12E-07	8.01E-05	down	LAD1
ENSSSCG00000022447	111	228	161	405	665	448	0.365527065	-1.451949864	0.000166813	0.010061497	down	F3
ENSSSCG00000022614	53	47	42	10	22	9	3.853247555	1.946074876	0.000449752	0.020735872	up	DQX1
ENSSSCG00000022689	337	303	569	113	68	150	4.241954045	2.084728992	2.10E-08	6.63E-06	up	GADD45B
ENSSSCG00000022891	47	90	61	18	4	6	7.788443706	2.961335076	1.43E-05	0.001428638	up	MYMK
ENSSSCG00000024223	17	76	98	75	217	2712	0.082745192	-3.595180711	0.000904157	0.034254707	down	ARHGEF16
ENSSSCG00000024236	5	9	9	68	47	52	0.154425353	-2.695018469	2.40E-07	5.03E-05	down	MMP17

ENSSSCG00000024393	4	5	11	55	27	313	0.064330971	-3.958342728	1.69E-05	0.00160453	down	B3GNT5
ENSSSCG00000024596	224	245	320	1313	1033	579	0.293815888	-1.767015682	4.36E-05	0.003592034	down	NOCT
ENSSSCG00000026044	3863	3936	2661	1487	1222	1966	2.587879129	1.371770236	7.05E-09	2.40E-06	up	FDFT1
ENSSSCG00000026068	31	25	34	9	10	6	4.042994155	2.015424118	0.000236496	0.012866303	up	GRHL1
ENSSSCG00000026149	1021	1129	738	355	402	294	3.06510766	1.615937749	2.79E-07	5.75E-05	up	PKLR
ENSSSCG00000026297	299	288	162	141	89	121	2.426639086	1.278959552	0.000589399	0.025077707	up	KLB
ENSSSCG00000026427	2347	1302	1406	645	481	746	3.12828738	1.645373052	4.70E-07	8.88E-05	up	RORC
ENSSSCG00000027232	34	25	45	160	72	108	0.34473505	-1.536440108	0.001171268	0.041057765	down	ENSSSCG00000027232
ENSSSCG00000028010	2181	1086	1108	504	339	707	3.325956918	1.733769481	1.15E-05	0.00124517	up	ABHD3
ENSSSCG00000028056	2337	2854	3203	1167	917	1145	2.943064345	1.557319085	1.44E-14	1.51E-11	up	ZFP36
ENSSSCG00000028124	51	41	71	158	116	1102	0.151823467	-2.719533289	0.000368406	0.018024083	down	SNRPN
ENSSSCG00000028185	91	164	101	268	215	349	0.49109288	-1.025932189	0.000573693	0.02477079	down	FGD3
ENSSSCG00000028322	7609	10769	8018	2958	2301	1764	4.142629281	2.050546721	1.12E-09	4.76E-07	up	BTG2
ENSSSCG00000028517	6930	4810	4265	2287	2426	1214	2.971476696	1.571180067	0.000277351	0.014621115	up	SC5D
ENSSSCG00000028572	433	478	541	253	136	326	2.360994584	1.239394732	4.59E-05	0.003756685	up	PER3
ENSSSCG00000028695	15	14	19	3	3	3	6.07716946	2.603399522	0.00044831	0.020735872	up	ENSSSCG00000028695
ENSSSCG00000028821	696	496	684	230	118	95	4.665366874	2.221990536	1.36E-06	0.000222523	up	SASS6
ENSSSCG00000028923	21	25	48	455	354	483	0.08270074	-3.595955947	1.65E-23	7.48E-20	down	SCNN1B
ENSSSCG00000028944	11	20	17	42	62	114	0.259086613	-1.948493623	5.35E-05	0.004181765	down	ENSSSCG00000028944
ENSSSCG00000029066	4661	3558	3307	667	618	333	7.830896509	2.969177482	5.57E-13	4.74E-10	up	ENSSSCG00000029066
ENSSSCG00000029070	72	84	65	212	184	177	0.433205131	-1.206877766	2.76E-05	0.00247333	down	PAFAH1B3
ENSSSCG00000029212	125	125	132	265	282	476	0.435529569	-1.199157425	7.45E-08	1.84E-05	down	GDE1
ENSSSCG00000029596	128	158	157	76	62	42	2.717568798	1.442316559	0.0001688	0.010069535	up	ENSSSCG00000029596
ENSSSCG00000029658	16	13	14	108	113	39	0.179169231	-2.480605194	7.30E-05	0.005195501	down	ENSSSCG00000029658
ENSSSCG00000029790	2579	6535	2653	1065	1034	1508	3.727836248	1.898338488	1.96E-06	0.000317914	up	PCYOX1
ENSSSCG00000029944	2506	5917	6300	1399	1649	1587	3.557532002	1.830876735	8.43E-07	0.00014704	up	FASN
ENSSSCG00000029990	31	122	79	633	127	922	0.162514694	-2.621357931	0.000214454	0.012064561	down	DEFB1
ENSSSCG00000030150	2457	2628	1323	1057	1193	843	2.30837127	1.20687528	0.001461832	0.04756549	up	UGDH
ENSSSCG00000030269	16	24	30	7	4	9	4.026820397	2.009641127	0.000960043	0.035597642	up	ENSSSCG00000030269
ENSSSCG00000030882	11	24	17	55	114	95	0.222550913	-2.167792674	9.83E-06	0.001096241	down	ENSSSCG00000030882
ENSSSCG00000030901	18	8	10	0	0	0	74.9947409	6.228717523	6.71E-05	0.004882894	up	ENSSSCG00000030901
ENSSSCG00000031255	295	413	205	141	154	197	2.126897213	1.088750314	0.000553921	0.024069902	up	ENSSSCG00000031255
ENSSSCG00000031306	30	28	17	1	2	0	28.06778896	4.810843515	4.86E-06	0.000642063	up	ENSSSCG00000031306
ENSSSCG00000031356	226	455	435	163	135	266	2.294026537	1.19788208	0.00046443	0.021268383	up	HES1
ENSSSCG00000031449	98	142	90	48	45	19	3.203583604	1.679686641	0.001265754	0.043255072	up	SLC27A5
ENSSSCG00000031616	375	1067	266	13	30	15	32.43003575	5.019258709	5.08E-15	6.91E-12	up	FOSB
ENSSSCG00000031617	347	478	313	1131	951	2173	0.31536201	-1.664919218	4.63E-08	1.21E-05	down	SFT2D2
ENSSSCG00000031712	0	1	2	20	6	32	0.061202868	-4.030256936	0.001084688	0.03883938	down	MFAP5
ENSSSCG00000031735	362	1482	894	290	377	241	3.298850275	1.7219633	0.001078917	0.038821043	up	ENSSSCG00000031735
ENSSSCG00000031904	1197	964	899	289	449	183	3.643170842	1.86519465	2.79E-05	0.002477562	up	UGT8
ENSSSCG00000031912	4963	7087	5012	1381	1549	1842	4.077621419	2.027727836	3.60E-25	2.45E-21	up	ENSSSCG00000031912
ENSSSCG00000032115	4770	4324	2657	1169	1091	1237	3.829329202	1.937091692	2.62E-11	1.88E-08	up	OSGIN1
ENSSSCG00000032282	535	1202	902	390	267	205	3.346645142	1.742715584	6.48E-05	0.004762514	up	ACP5
ENSSSCG00000032320	2876	1280	1596	341	679	361	4.67160605	2.223918619	4.46E-06	0.000612231	up	TCIM
ENSSSCG00000032416	192	225	803	96	82	108	4.83435088	2.273322189	9.54E-05	0.006622937	up	RGS1
ENSSSCG00000032433	30	29	44	8	3	4	7.740035433	2.952340171	3.63E-06	0.000525341	up	PTCHD1
ENSSSCG00000032607	723	930	810	1978	1252	5812	0.333006937	-1.586375864	0.00082097	0.031721619	down	ALPL
ENSSSCG00000032768	2951	3511	3159	1171	1500	509	3.265637074	1.707364466	0.000359184	0.017700224	up	EBP
ENSSSCG00000032777	1487	1208	450	175	251	54	7.06185709	2.820047626	4.70E-05	0.003826415	up	CXCL9
ENSSSCG00000032816	54	36	34	10	19	19	2.980079766	1.575350947	0.001278106	0.043350434	up	ENSSSCG00000032816
ENSSSCG00000033015	5	8	7	20	21	64	0.229077288	-2.126093665	0.001543161	0.049403199	down	B3GNT7
ENSSSCG00000033701	131	66	27	2	5	0	35.10237298	5.133496657	6.61E-07	0.000118224	up	ENSSSCG00000033701
ENSSSCG00000033733	670	727	674	1252	1193	2453	0.498213395	-1.005164285	3.06E-05	0.002669564	down	ENSSSCG00000033733
ENSSSCG00000033858	69	96	102	40	43	44	2.381470839	1.251852883	4.17E-05	0.00349178	up	ENSSSCG00000033858
ENSSSCG00000034340	5	3	13	25	25	168	0.121848332	-3.036841597	0.001128384	0.040070877	down	ENSSSCG00000034340
ENSSSCG00000034360	1363	1179	949	603	700	486	2.177667589	1.12278375	0.000446209	0.020712948	up	CELSR2
ENSSSCG00000034632	383	190	74	29	41	90	4.912658607	2.296503986	0.000563578	0.024411558	up	PDXP

ENSSSCG00000034817	339	354	208	77	87	97	3.946175813	1.980455234	1.08E-10	5.89E-08	up	ENSSSCG00000034817
ENSSSCG00000034976	7504	6213	5702	2921	2997	1458	2.883287426	1.527714662	0.000236256	0.012866303	up	UGT2A3
ENSSSCG00000034993	411	547	491	205	202	246	2.527867727	1.337920975	1.02E-14	1.16E-11	up	NREP
ENSSSCG00000035020	10	15	14	84	95	88	0.16466309	-2.602410892	3.62E-11	2.46E-08	down	STK32B
ENSSSCG00000035077	1012	1449	1261	348	723	322	2.928399381	1.550112325	0.000337241	0.017051365	up	INHBA
ENSSSCG00000035114	1805	1451	1464	903	701	739	2.273119908	1.184673789	4.33E-06	0.000600923	up	CLDN12
ENSSSCG00000035152	487	573	555	358	173	369	2.06256199	1.04443748	0.000767404	0.03060839	up	TEF
ENSSSCG00000035212	254	434	692	181	167	199	2.848609252	1.510257739	0.000104374	0.007097983	up	KLF6
ENSSSCG00000035243	21	22	39	166	77	162	0.231955356	-2.108080935	4.33E-06	0.000600923	down	RAB27B
ENSSSCG00000035249	691	2193	1368	549	292	350	3.94311404	1.979335436	4.18E-05	0.00349178	up	GADD45G
ENSSSCG00000035347	47012	16001	10865	4359	4000	3592	7.057998733	2.819259171	9.98E-07	0.000169631	up	ENSSSCG00000035347
ENSSSCG00000035379	299	917	894	245	272	266	3.010602132	1.59005206	0.000311278	0.016036687	up	JCHAIN
ENSSSCG00000035561	59	70	52	133	141	163	0.47242234	-1.081850907	9.53E-06	0.00107127	down	ENSSSCG00000035561
ENSSSCG00000035617	26	56	38	393	594	1272	0.063212158	-3.983654128	1.00E-18	1.94E-15	down	ENSSSCG00000035617
ENSSSCG00000035663	100	85	120	321	242	229	0.430738671	-1.215115244	0.00021481	0.012064561	down	ENSSSCG00000035663
ENSSSCG00000035863	240	280	365	1903	1302	1200	0.222752183	-2.166488525	1.71E-10	8.96E-08	down	PLIN2
ENSSSCG00000035891	6	4	6	20	17	94	0.152490693	-2.713206898	0.001056142	0.03820369	down	ADGRB1
ENSSSCG00000035987	721	1001	802	439	461	471	2.0786962	1.055678925	1.59E-07	3.50E-05	up	EHD3
ENSSSCG00000036206	1176	701	785	369	368	274	2.950730956	1.561072384	1.44E-05	0.001433186	up	DIPK2A
ENSSSCG00000036224	305	1873	2576	298	336	238	5.963569087	2.576176016	0.000138002	0.008730055	up	ENSSSCG00000036224
ENSSSCG00000036411	4121	2584	2156	1254	1532	1948	2.174447056	1.120648582	0.000485047	0.021844795	up	ACOT13
ENSSSCG00000036494	3826	2678	2131	1459	1499	1205	2.332431344	1.221834615	0.000336762	0.017051365	up	ERG28
ENSSSCG00000036589	51	33	62	11	13	6	5.41295127	2.4364154	2.36E-05	0.002148361	up	BEGAIN
ENSSSCG00000036613	69	51	92	11	4	18	7.572285014	2.920728714	1.31E-07	2.96E-05	up	POLQ
ENSSSCG00000036679	1996	1881	2210	1140	1293	823	2.069353976	1.049180448	0.000867609	0.033161335	up	SORBS2
ENSSSCG00000036695	24	10	8	66	82	50	0.237835908	-2.071961546	0.00075041	0.030344922	down	IGF2BP3
ENSSSCG00000036748	0	0	0	7	11	45	0.010903786	-6.519027077	7.90E-05	0.005597929	down	ENSSSCG00000036748
ENSSSCG00000036785	32	62	52	499	464	222	0.133252127	-2.907769534	4.66E-09	1.71E-06	down	ENSSSCG00000036785
ENSSSCG00000036975	441	371	287	189	236	189	2.018334874	1.013165561	0.001038464	0.037866354	up	PSRC1
ENSSSCG00000037009	59	741	1757	59	70	74	14.08956257	3.816554917	1.96E-05	0.001813868	up	ENSSSCG00000037009
ENSSSCG00000037665	13	52	13	0	0	3	31.53361958	4.978818874	0.000215549	0.012064561	up	ENSSSCG00000037665
ENSSSCG00000037775	1737	5218	3980	1562	1737	1582	2.492948497	1.317853078	0.001246348	0.042807023	up	ENSSSCG00000037775
ENSSSCG00000037910	1049	2330	1853	817	906	605	2.468544849	1.303660856	0.000789768	0.031119966	up	DUSP6
ENSSSCG00000037954	61	32	25	5	0	1	22.14365601	4.468821531	1.07E-05	0.001168515	up	CAVIN4
ENSSSCG00000038062	76	53	77	25	16	14	4.201044651	2.07074812	8.13E-06	0.000961096	up	SAMSN1
ENSSSCG00000038207	0	0	0	2	5	57	0.011305567	-6.466822801	0.001458041	0.047555902	down	ssc-mir-503
ENSSSCG00000038549	2512	4712	2704	1531	1652	1888	2.218041022	1.149286048	5.29E-05	0.004156709	up	ZFP36L2
ENSSSCG00000038562	227	330	729	80	111	125	4.63236873	2.211750094	2.79E-06	0.000435958	up	RND1
ENSSSCG00000038580	422	321	641	166	169	253	2.723407158	1.445412686	6.77E-06	0.000819019	up	ENSSSCG00000038580
ENSSSCG00000038660	10048	8275	6026	4797	4617	4037	2.036359427	1.025992226	0.000794302	0.031119966	up	HAAO
ENSSSCG00000038663	52	60	69	752	748	478	0.100786957	-3.310619151	1.83E-19	4.14E-16	down	ENSSSCG00000038663
ENSSSCG00000038693	12	10	6	31	33	208	0.130803382	-2.934528257	0.000355151	0.017565116	down	RAB19
ENSSSCG00000038719	26	176	143	16	23	44	4.84044786	2.275140538	0.001492708	0.048454225	up	ENSSSCG00000038719
ENSSSCG00000038946	143	125	144	20	40	29	5.249266167	2.392115752	5.46E-11	3.23E-08	up	SLC17A8
ENSSSCG00000039147	69	72	58	189	194	439	0.28772263	-1.797249396	1.72E-07	3.71E-05	down	ENSSSCG00000039147
ENSSSCG00000039333	0	4	6	28	12	145	0.067790753	-3.882767693	0.000952917	0.035508561	down	RFX6
ENSSSCG00000039443	0	2	2	19	27	17	0.070283684	-3.830666368	0.000146773	0.009118895	down	ENSSSCG00000039443
ENSSSCG00000039760	425	321	340	215	101	92	2.942633317	1.557107779	0.000763414	0.030538792	up	PRR16
ENSSSCG00000039815	88	95	92	395	295	640	0.242204086	-2.04570789	4.79E-14	4.35E-11	down	ENSSSCG00000039815
ENSSSCG00000039825	9	7	10	64	40	24	0.222254747	-2.169713859	0.001034773	0.037855467	down	ENSSSCG00000039825
ENSSSCG00000040085	54	9	8	1	0	0	77.19639205	6.270461516	0.000152302	0.009373091	up	ENSSSCG00000040085
ENSSSCG00000040260	1	4	2	14	17	88	0.073155001	-3.77289969	0.000223911	0.012481208	down	ENSSSCG00000040260
ENSSSCG00000040638	674	649	403	156	151	56	5.159269415	2.367166785	6.60E-06	0.000810385	up	DIO2
ENSSSCG00000040678	51	64	192	23	25	26	4.677616214	2.225773497	0.000234156	0.012841754	up	TYMS
ENSSSCG00000040735	2022	2382	2349	3484	4223	10335	0.449140338	-1.154761798	0.000960542	0.035597642	down	DDAH1
ENSSSCG00000040849	48	39	94	9	15	12	5.692377459	2.509031329	7.26E-06	0.000865703	up	ENSSSCG00000040849
ENSSSCG00000040925	670	651	323	213	151	68	4.133493138	2.047361492	0.000268715	0.014332513	up	ENSSSCG00000040925

ENSSSCG00000040986	3	22	19	0	0	0	89.20133438	6.478993387	0.000173811	0.010265473	up	ENSSSCG00000040986

Table S3 DEG for enrichment

source	term_name	term_id	djusted_p_valu	negative_log10_of_adjusted_p_value	Fold_enrichment	term_size	query_size	intersection_size	ffective_domain_siz	intersections
GO:BP	steroid metabolic process	GO:0008202	2.16E-05	4.665740464	6.482451763	167	216	15	15589	RDH16,BMP6,CYP1A1,RORA,SQLE,MVK,EGR1,CYP51A1,LSS,CLCN2,FDFT1,SC5D,SLC27A5,EBP,ERG28
GO:BP	steroid biosynthetic process	GO:0006694	2.86E-05	4.543115128	8.574807481	101	216	12	15589	BMP6,SQLE,MVK,EGR1,CYP51A1,LSS,CLCN2,FDFT1,SC5D,SLC27A5,EBP,ERG28
GO:BP	small molecule metabolic process	GO:0044281	9.06E-05	4.042695811	2.444511649	1240	216	42	15589	PMM1,ENSSSCG00000000576,IGF1,PPP1R3G,BMP6,CYP1A1,APRT,ACAT2,MOXD1,RORA,HACD3,KYAT1,SQLE,MVK,MAT1A,ALOX5,SCD,PFKFB3,EGR1,CYP51A1,ATF3,ATIC,AGXT,GCK,LSS,CLCN2,FDFT1,PKLR,SC5D,GDE1,PCYOX1,UGDH,SLC27A5,EBP,PDXP,UGT2A3,ENSSSCG00000035347,ERG28,HAAO,ENSSSCG00000040085,TYMS,DDAH1
GO:BP	sterol biosynthetic process	GO:0016126	9.76E-05	4.010538015	14.80436847	39	216	8	15589	SQLE,MVK,CYP51A1,LSS,FDFT1,SC5D,EBP,ERG28
GO:BP	lipid metabolic process	GO:0006629	0.000294142	3.53144317	2.715681617	877	216	33	15589	RDH16,PIK3C2G,BMP6,PLA2G7,PLIN1,CYP1A1,TTC7B,ENSSSCG00000002825,ACAT2,RORA,HACD3,SQLE,ID2,MVK,LIPM,SCD,ENSSSCG00000013900,EGR1,CYP51A1,LSS,CLCN2,FDFT1,ABHD3,SC5D,ENSSSCG00000029066,GDE1,ENSSSCG00000030269,SLC27A5,EBP,ENSSSCG00000035347,ERG28,ENSSSCG00000036785,ENSSSCG00000040085

GO:BP	secondary alcohol biosynthetic process	GO:1902653	0.000669792	3.174059953	14.8587963	34	216	7	15589	MVK,CYP51A1,LSS,FDFT1,SC5D,EBP,ERG28
GO:BP	small molecule biosynthetic process	GO:0044283	0.004217234	2.374972264	3.372490481	428	216	20	15589	BMP6,MOXD1,HACD3,MVK,ALOX5,SCD,EGR1,CYP51A1,ATF3,AGXT,GCK,LSS,CLCN2,FDFT1,SC5D,UGDH,SLC27A5,EBP,ERG28,HAAO
GO:BP	organic hydroxy compound metabolic process	GO:1901615	0.005424824	2.265614354	3.786765546	324	216	17	15589	BMP6,CYP1A1,MOXD1,SQLE,MVK,COMT,CYP51A1,LSS,CLCN2,FDFT1,SC5D,GDE1,SLC27A5,EBP,PDXP,ERG28,DIO2
GO:BP	cholesterol biosynthetic process	GO:0006695	0.005709544	2.243398587	14.43425926	30	216	6	15589	MVK,CYP51A1,LSS,FDFT1,SC5D,EBP
GO:BP	organic hydroxy compound biosynthetic process	GO:1901617	0.006237312	2.205002549	5.248821549	165	216	12	15589	BMP6,MOXD1,SQLE,MVK,CYP51A1,LSS,CLCN2,FDFT1,SC5D,SLC27A5,EBP,ERG28
GO:BP	alcohol biosynthetic process	GO:0046165	0.006732321	2.171835207	6.443865741	112	216	10	15589	BMP6,MOXD1,MVK,CYP51A1,LSS,CLCN2,FDFT1,SC5D,EBP,ERG28
GO:BP	regulation of B cell activation	GO:0050864	0.010762694	1.968078991	6.984318996	93	216	9	15589	CDKN1A,TNFAIP3,ID2,ENSSSCG00000036224,ENSSSCG00000037775,SAMSN1,ZFP36L2,ENSSSCG00000038719,ENSSSCG00000040849
GO:BP	phagocytosis, recognition	GO:0006910	0.015924454	1.797935455	17.18364198	21	216	5	15589	ADGRB1,ENSSSCG00000036224,ENSSSCG00000037775,ENSSSCG00000038719,ENSSSCG00000040849
GO:BP	drug metabolic process	GO:0017144	0.024808584	1.605398015	3.542149511	326	216	16	15589	CYP1A1,APRT,MOXD1,RORA,COMT,MAT1A,EGR1,MMP3,AGXT,SLC27A5,ACP5,PDXP,ENSSSCG00000035347,HAAO,ENSSSCG00000040085,DDAH1

GO:BP	response to oxygen-containing compound	GO:1901700	0.025875889	1.587104727	2.41722045	836	216	28	15589	TIMELESS,IGF1,BMP6,FOS,TNFAIP3,RORA,SLC2A8,VCAM1,MMP9,DMTN,KCNMA1,SCD,CRY2,ENSSSCG00000013852,ENSSSCG00000013900,EGR1,CACNA2D1,GCK,PRKCA,GLP2R,NOCT,PKLR,RORC,ZFP36,ACP5,CXCL9,PDXP,RFX6
GO:BP	sterol metabolic process	GO:0016125	0.033934061	1.469364164	7.041102078	82	216	8	15589	SQLE,MVK,CYP51A1,LSS,FDFT1,SC5D,EBP,ERG28
GO:BP	alcohol metabolic process	GO:0006066	0.03463129	1.460531335	4.115030052	228	216	13	15589	BMP6,CYP1A1,MOXD1,SQLE,MVK,CYP51A1,LSS,CLCN2,FDFT1,SC5D,GDE1,EBP,ERG28
GO:BP	secondary alcohol metabolic process	GO:1902652	0.044219845	1.354382782	6.792592593	85	216	8	15589	SQLE,MVK,CYP51A1,LSS,FDFT1,SC5D,EBP,ERG28
KEGG	Metabolic pathways	KEGG:01100	1.14E-05	4.941374595	2.070907678	1327	127	48	7271	PMM1,RDH16,PIK3C2G,PLA2G7,CYP1A1,APRT,GPT2,CYP4A24,ACAT2,HACD3,KYAT1,SQLE,SELENBP1,MVK,COMT,MAT1A,OGDHL,ALOX5,SCD,PFKFB3,CYP51A1,ATIC,AGXT,INMT,GCK,SLC45A2,ND6,LSS,B3GNT5,FDFT1,PKLR,SC5D,ENSSSCG00000029066,PAFAH1B3,FASN,UGDH,SLC27A5,UGT8,ACP5,ALPL,EBP,PDXP,UGT2A3,ENSSSCG00000035347,ENSSSCG00000036785,ENSSSCG00000038580,HAAO,TYMS
KEGG	Steroid biosynthesis	KEGG:00100	4.86E-05	4.312948018	18.079569	19	127	6	7271	SQLE,CYP51A1,LSS,FDFT1,SC5D,EBP
KEGG	PPAR signaling pathway	KEGG:03320	0.000368762	3.433253786	7.05846187	73	127	9	7271	PLIN1,CYP4A24,SCD,ENSSSCG00000010992,PLIN4,PLIN5,MMP3,SLC27A5,PLIN2
KEGG	Terpenoid backbone biosynthesis	KEGG:00900	0.036127213	1.442165538	10.90513686	21	127	4	7271	ACAT2,MVK,ENSSSCG00000029066,PCYOX1

Table S4 DEP list

Accession	gene name	Description	TP_1	TP_2	TP_3	YY_1	YY_2	YY_3	Fold Change	pvalue
Q66LN4	#N/A	Enoyl-Coenzyme A hydratase/3-hydroxyacyl Coenzyme A (Fragment) OS=Sus scrofa OX=9823 PE=2 SV=1	207.3	107.3	124.6	45.2	65.8	49.8	2.731343284	0.042064822
F1RUQ4	LOC100516628	UDP-glucuronosyltransferase OS=Sus scrofa OX=9823 GN=LOC100516628 PE=1 SV=3	164.1	107.1	159	47.1	68.5	54.2	2.533568905	0.010779082
A0A1S6L948	PLE-C4	Carboxylic ester hydrolase OS=Sus scrofa OX=9823 GN=PLE-C4 PE=3 SV=1	132.1	110.3	184.6	49.7	50.3	73	2.468208092	0.02222624
F1SL81	LOC100512656	Uncharacterized protein OS=Sus scrofa OX=9823 GN=LOC100512656 PE=1 SV=3	140.4	123.5	155.4	59.5	72.7	48.5	2.320420587	0.002345384
K7ZPU8	IGHG5-1	IgG heavy chian constant region (Fragment) OS=Sus scrofa OX=9823 GN=IGHG5-1 PE=4 SV=1	134.6	177.1	105.9	70.2	54	58.1	2.290729567	0.020987783
L8B0V2	IGHG	IgG heavy chain OS=Sus scrofa OX=9823 GN=IGHG PE=2 SV=1	161.8	126.4	120.5	61.9	62.7	66.8	2.135318704	0.005063432

I3LM80	DHCR24	Uncharacterized protein OS=Sus scrofa OX=9823 GN=DHCR24 PE=4 SV=1	141.5	113.2	129.2	75.7	66.5	73.8	1.777314815	0.002951607
I3L9F5	PCYOX1	Prenylcysteine oxidase 1 OS=Sus scrofa OX=9823 GN=PCYOX1 PE=1 SV=2	164.6	109	107.2	71	74.3	73.9	1.737226277	0.046173852
F1RL06	LOC100523213	Uncharacterized protein OS=Sus scrofa OX=9823 GN=LOC100523213 PE=1 SV=3	154.2	133.6	92.9	74.1	76.3	68.9	1.735978112	0.041336716
I3LF61	LOC110255237	Uncharacterized protein OS=Sus scrofa OX=9823 GN=LOC110255237 PE=1 SV=2	108.4	134.9	134	74.5	67.1	81.1	1.694207454	0.005776278
F1SFI5	HRG	Uncharacterized protein OS=Sus scrofa OX=9823 GN=HRG PE=1 SV=2	158.5	106.5	112.1	63.1	80.5	79.3	1.69179004	0.041861308
F1SGC6	NDUFB5	Uncharacterized protein OS=Sus scrofa OX=9823 GN=NDUFB5 PE=1 SV=1	116.6	153.4	104.9	73.8	79.8	71.5	1.665482008	0.02806333
A0A0K0TQQ8	PPARD	Peroxisome proliferator-activated receptor delta OS=Sus scrofa OX=9823 GN=PPARD PE=2 SV=1	115.6	149.4	107.1	80.5	78.1	69.2	1.633450395	0.022795517

A0A287BMK6	LOC110259329	Uncharacterized protein OS=Sus scrofa OX=9823 GN=LOC110259329 PE=3 SV=1	119.2	142.5	107.2	75.1	79.9	76	1.596969697	0.011768056
F1S6K5	APRT	Adenine phosphoribosyltransferase OS=Sus scrofa OX=9823 GN=APRT PE=3 SV=1	143	101.9	124	73.6	75.5	82.1	1.595588235	0.019483119
L8AXL9	IGHG	IgG heavy chain OS=Sus scrofa OX=9823 GN=IGHG PE=2 SV=1	112.8	142.9	105.9	75.8	85.9	76.7	1.516778523	0.025406926
F1SJ26	CYP1A2	Cytochrome P450 OS=Sus scrofa OX=9823 GN=CYP1A2 PE=1 SV=1	124.6	137	96.8	75.1	83.7	82.8	1.483443709	0.03313074
F1RSV0	URAD	Uncharacterized protein OS=Sus scrofa OX=9823 GN=URAD PE=4 SV=1	134	94.4	127.9	75.2	80	88.5	1.462043496	0.04379129
F1RL04	PPM1F	Protein phosphatase, Mg2+/Mn2+ dependent 1F OS=Sus scrofa OX=9823 GN=PPM1F PE=1 SV=2	127.9	98.2	127.8	70.8	94.4	81	1.437449228	0.040428341
L8AXK8	IGHG	IgG heavy chain OS=Sus scrofa OX=9823 GN=IGHG PE=2 SV=1	118.3	122.8	106.4	83.1	76.5	92.9	1.376237624	0.009754166

A0A2C9F366	HSPB1	Heat shock protein beta-1 OS=Sus scrofa OX=9823 GN=HSPB1 PE=1 SV=1	100.9	128.4	116.9	84.2	85.9	83.7	1.364066194	0.018315123
A0A287AE42	ECH1	Enoyl-CoA hydratase 1 OS=Sus scrofa OX=9823 GN=ECH1 PE=1 SV=1	101.9	109.1	134.9	88.6	77.2	88.3	1.361275089	0.045903405
L8B0R9	IGHG	IgG heavy chain OS=Sus scrofa OX=9823 GN=IGHG PE=2 SV=1	112	128.2	105	96.4	75.2	83.2	1.354788069	0.03106364
F1STC5	#N/A	Uncharacterized protein OS=Sus scrofa OX=9823 PE=1 SV=4	119.9	126.3	97	93	76.5	87.3	1.336448598	0.046655744
F1RX37	FGB	Fibrinogen beta chain OS=Sus scrofa OX=9823 GN=FGB PE=1 SV=2	104.2	107.2	130.1	84.2	80	94.3	1.321083172	0.039844391
A0A287BAY9	ALB	Serum albumin OS=Sus scrofa OX=9823 GN=ALB PE=1 SV=1	116.6	114.7	109.4	90.6	89.2	79.4	1.314429012	0.002764214
F1SCC9	LOC106504545	Uncharacterized protein OS=Sus scrofa OX=9823 GN=LOC106504545 PE=1 SV=2	114.1	115.9	109.5	86.4	90.9	83.2	1.303262956	0.00085482
I3LJ21	UBE2G2	Ubiquitin conjugating enzyme E2 G2 OS=Sus scrofa OX=9823 GN=UBE2G2 PE=1 SV=2	107.8	114.2	117.2	81.8	97.8	81.2	1.300613497	0.012820331

I3LRD5	EIF4H	Uncharacterized protein OS=Sus scrofa OX=9823 GN=EIF4H PE=1 SV=1	111.4	118	108.3	87	93.7	81.6	1.28745711	0.005121075
A0A287AMA6	SORBS2	Sorbin and SH3 domain-containing protein 2 OS=Sus scrofa OX=9823 GN=SORBS2 PE=1 SV=1	108.4	113.7	115.1	86.7	94.2	81.8	1.283593453	0.003896687
F1S8W1	COX15	Cytochrome c oxidase assembly homolog COX15 OS=Sus scrofa OX=9823 GN=COX15 PE=1 SV=3	119.8	104.8	108.5	88.7	92.2	86.1	1.247565543	0.01043916
F1RYZ1	CD151	Tetraspanin OS=Sus scrofa OX=9823 GN=CD151 PE=1 SV=1	115.8	119.4	97.6	87.3	90.3	89.5	1.24597529	0.032357379
F1RZZ3	FN3KRP	Fructosamine 3 kinase related protein OS=Sus scrofa OX=9823 GN=FN3KRP PE=1 SV=2	116.3	95.9	118.9	90.9	91.2	86.9	1.230855019	0.049004318
P05027	ATP1B1	Sodium/potassium-transporting ATPase subunit beta-1 OS=Sus scrofa OX=9823 GN=ATP1B1 PE=1 SV=2	108.4	118.8	102.8	96.1	91.5	82.5	1.221769715	0.031606692

I3LMV8	ABCB8	ATP binding cassette subfamily B member 8 OS=Sus scrofa OX=9823 GN=ABCB8 PE=1 SV=2	110.7	109.7	108.9	88.4	80.8	101.4	1.216925351	0.031639038
F1RIA7	TMEM109	Transmembrane protein 109 OS=Sus scrofa OX=9823 GN=TMEM109 PE=1 SV=1	120	108.3	100.6	89.3	95.8	85.9	1.213653137	0.038312076
A0A286ZZ11	CYP2C36	Uncharacterized protein OS=Sus scrofa OX=9823 GN=CYP2C36 PE=3 SV=1	117.3	102.8	108.6	89.8	97.7	83.8	1.211573903	0.03040094
A0A287AP73	ITIH3	Inter-alpha-trypsin inhibitor heavy chain 3 OS=Sus scrofa OX=9823 GN=ITIH3 PE=1 SV=1	111.4	105.3	111.8	88.1	91.5	91.8	1.210390567	0.001400785
F1S0W9	NUP214	Nucleoporin 214 OS=Sus scrofa OX=9823 GN=NUP214 PE=1 SV=2	97.3	87.7	87.7	114.9	102.6	109.9	0.832926084	0.019064722
A0A287BCJ2	TTC36	Tetratricopeptide repeat domain 36 OS=Sus scrofa OX=9823 GN=TTC36 PE=1 SV=1	91.2	82.9	98.5	107.4	107.4	112.6	0.832620648	0.019381012
A0A173G6H3	#N/A	Septin 8 OS=Sus scrofa OX=9823 PE=2 SV=1	98.7	80.7	93.2	111.1	104.2	112.2	0.832366412	0.035879
A0A2S1PVW4	RIG-I	RIG-I OS=Sus scrofa OX=9823 GN=RIG-I PE=2 SV=1	90	91.6	91	121.9	103.3	102.3	0.832366412	0.045757011

F1SPF9	COPG1	Coatomer subunit gamma OS=Sus scrofa OX=9823 GN=COPG1 PE=1 SV=3	95.3	80.3	96.9	111.9	102.1	113.4	0.832315211	0.045223325
F1RK00	ZC3H13	Zinc finger CCCH-type containing 13 OS=Sus scrofa OX=9823 GN=ZC3H13 PE=4 SV=3	87.5	88.8	96.2	101.8	118.1	107.6	0.832061069	0.02878549
F1RKG8	PEBP1	Uncharacterized protein OS=Sus scrofa OX=9823 GN=PEBP1 PE=1 SV=1	94	84.6	93.9	105.8	115.3	106.5	0.831807082	0.013616651
A0A287A892	USP19	Ubiquitin specific peptidase 19 OS=Sus scrofa OX=9823 GN=USP19 PE=1 SV=1	96.3	87.4	88.7	106.3	108.5	112.7	0.831755725	0.005394001
A0A287ANK4	RCC2	Regulator of chromosome condensation 2 OS=Sus scrofa OX=9823 GN=RCC2 PE=1 SV=1	96.3	82.3	93.8	107.4	102.3	117.9	0.831501832	0.043189558
F1SLL6	MTREX	Mtr4 exosome RNA helicase OS=Sus scrofa OX=9823 GN=MTREX PE=1 SV=3	96	84.5	91.9	112	105.8	109.9	0.831248093	0.008535469
C0LZL0	FSCN1	Fascin OS=Sus scrofa OX=9823 GN=FSCN1 PE=1 SV=1	95.5	89.5	87.2	100.2	109.6	118.1	0.830131138	0.031683367
F1S3Q7	VNN1	Pantetheinase OS=Sus scrofa OX=9823 GN=VNN1 PE=4 SV=2	91.1	85.5	95.4	104.3	103.5	120.2	0.829268293	0.038531132

F1SKU1	CWC27	CWC27 spliceosome associated protein homolog OS=Sus scrofa OX=9823 GN=CWC27 PE=4 SV=3	93.7	83.3	94.9	102.1	111.2	114.8	0.828710759	0.023797054
F1S956	PGLS	6-phosphogluconolactonase OS=Sus scrofa OX=9823 GN=PGLS PE=1 SV=3	92.8	80.8	98.1	106.1	106.9	115.2	0.827848873	0.032919619
F1SQA9	SEC13	SEC13 homolog, nuclear pore and COPII coat complex component OS=Sus scrofa OX=9823 GN=SEC13 PE=1 SV=3	93.2	80.6	97.7	112.3	106.2	109.8	0.826987511	0.024979115
F1RHU2	AKT1S1	AKT1 substrate 1 OS=Sus scrofa OX=9823 GN=AKT1S1 PE=4 SV=2	91.9	92.7	86.8	109	105.1	114.5	0.82592818	0.004425956
F1RQN4	NUDT6	Nudix hydrolase 6 OS=Sus scrofa OX=9823 GN=NUDT6 PE=3 SV=2	97.9	81.5	91.8	111.5	104	113.3	0.824817518	0.026109736
A0A287BBG2	ATG5	Autophagy protein 5 OS=Sus scrofa OX=9823 GN=ATG5 PE=3 SV=1	91	89.9	89.7	103	107.6	118.9	0.82124431	0.014363498
F1SN58	SQOR	Uncharacterized protein OS=Sus scrofa OX=9823 GN=SQOR PE=1 SV=3	89.4	90.1	90.9	114.7	103.5	111.4	0.82038835	0.004157455

A0A287AZ25	SF1	Splicing factor 1 OS=Sus scrofa OX=9823 GN=SF1 PE=1 SV=1	93.2	82.2	95	114.4	100.9	114.3	0.82038835	0.030384047
I3LEI5	CES3	Carboxylic ester hydrolase OS=Sus scrofa OX=9823 GN=CES3 PE=1 SV=2	96.3	83.3	90.7	104.8	120.4	104.5	0.819836215	0.037490394
A0A287BPN5	UROC1	Urocanate hydratase 1 OS=Sus scrofa OX=9823 GN=UROC1 PE=1 SV=1	95	81.4	93.7	106.5	107.4	116	0.818732949	0.019588564
K7GS25	43714	Septin 6 OS=Sus scrofa OX=9823 GN=SEPT6 PE=1 SV=1	101.4	90.8	77.9	109.9	107.5	112.5	0.818732949	0.04548697
Q29387	EEF1G	Elongation factor 1-gamma (Fragment) OS=Sus scrofa OX=9823 GN=EEF1G PE=2 SV=2	94.5	78.8	96.6	111.6	108.4	109.9	0.818126705	0.024585481
A0A287AXJ7	ISYNA1	Inositol-3-phosphate synthase 1 OS=Sus scrofa OX=9823 GN=ISYNA1 PE=4 SV=1	97.1	90	82.8	106.8	111.5	111.7	0.817878788	0.010621339
M3VJZ7	LASP1	LIM and SH3 domain protein 1 OS=Sus scrofa OX=9823 GN=LASP1 PE=2 SV=1	102.3	81.7	85.9	107.9	114.3	107.9	0.817631021	0.039029389

F1RQ01	FERMT3	Fermitin family member 3 OS=Sus scrofa OX=9823 GN=FERMT3 PE=1 SV=2	94.3	85.5	90.1	108.6	106.3	115.3	0.817383404	0.005611635
F1RHM8	RANBP1	RAN binding protein 1 OS=Sus scrofa OX=9823 GN=RANBP1 PE=1 SV=1	91.4	82.9	95.4	109.5	103.4	117.3	0.81677771	0.020900221
A0A068F143	#N/A	Glutathione peroxidase OS=Sus scrofa OX=9823 PE=2 SV=1	88.7	87.5	93.5	106	109.4	114.9	0.816530427	0.003130337
F1S8X9	HOGA1	Uncharacterized protein OS=Sus scrofa OX=9823 GN=HOGA1 PE=1 SV=3	94.1	82	93.4	109.8	104.2	116.4	0.815677966	0.018314026
F1RVH4	COMMD3	COMM domain containing 3 OS=Sus scrofa OX=9823 GN=COMMD3 PE=1 SV=1	91.7	80.3	97.4	104.6	118.5	107.4	0.815128593	0.036373718
A0A286ZKH3	SPR	Uncharacterized protein OS=Sus scrofa OX=9823 GN=SPR PE=1 SV=1	98.5	77.9	92.9	104.1	114.7	111.8	0.814579552	0.041769229
A0A287B247	C5orf51	Chromosome 5 open reading frame 51 OS=Sus scrofa OX=9823 GN=C5orf51 PE=1 SV=1	93.6	77.6	98.1	106.2	108.1	116.3	0.814579552	0.04238104

F1SA56	FNTB	Protein farnesyltransferase subunit beta OS=Sus scrofa OX=9823 GN=FNTB PE=1 SV=3	91.9	84.2	93.2	106.7	105.8	118.2	0.814333233	0.013769861
E7EI20	ARHGDIA	Rho GDP dissociation inhibitor alpha OS=Sus scrofa OX=9823 GN=ARHGDIA PE=2 SV=1	97.2	76	96.1	110.8	109.1	110.9	0.814087062	0.041372776
A0A286ZRU9	SERPINH1	Uncharacterized protein OS=Sus scrofa OX=9823 GN=SERPINH1 PE=1 SV=1	87.4	88.9	92.9	114.4	100.5	115.8	0.814030844	0.016413401
F1SJI1	SCLY	Selenocysteine lyase OS=Sus scrofa OX=9823 GN=SCLY PE=1 SV=3	101.7	76.7	90.8	110.7	108.9	111.2	0.813784764	0.047618665
A0A287AHD3	EML4	Echinoderm microtubule associated protein like 4 OS=Sus scrofa OX=9823 GN=EML4 PE=1 SV=1	94.3	81	93.8	115.1	103.2	112.6	0.813236627	0.022007186
D0G6X9	HPGD	15-hydroxyprostaglandin dehydrogenase OS=Sus scrofa OX=9823 GN=HPGD PE=1 SV=1	97.8	89.4	81.8	106.5	105.1	119.4	0.812688822	0.033329048

F1SDP2	LHPP	Phospholysine phosphohistidine inorganic pyrophosphate phosphatase OS=Sus scrofa OX=9823 GN=LHPP PE=1 SV=3	97.5	80.6	90.9	110.3	117.7	103	0.812688822	0.033477872
A0A286ZUU8	XPO7	Exportin 7 OS=Sus scrofa OX=9823 GN=XPO7 PE=1 SV=1	90.1	85.4	93.4	104	105.2	121.9	0.812141347	0.029101377
Q0GAA4	SLA-1	MHC class I antigen OS=Sus scrofa OX=9823 GN=SLA-1 PE=2 SV=1	82.6	100	86.2	116.8	102.3	112.1	0.811594203	0.037851993
I3LN84	LXN	Latexin OS=Sus scrofa OX=9823 GN=LXN PE=1 SV=1	94.9	79.7	94.2	108.4	102.9	119.9	0.811594203	0.041860686
F1RGQ6	ANKFY1	Ankyrin repeat and FYVE domain containing 1 OS=Sus scrofa OX=9823 GN=ANKFY1 PE=1 SV=2	88.6	83.6	96.5	107.2	104.3	119.9	0.810802655	0.026446531
I3LLP1	ABCA8	ATP binding cassette subfamily A member 8 OS=Sus scrofa OX=9823 GN=ABCA8 PE=1 SV=2	97	87	84.6	101.7	116.5	113.2	0.810500905	0.023545477
F1S0N2	ACLY	ATP-citrate synthase OS=Sus scrofa OX=9823 GN=ACLY PE=1 SV=3	89.9	83.2	95.5	106.5	107.5	117.4	0.810500905	0.013603428

I3LP11	KHDRBS1	KH RNA binding domain containing, signal transduction associated 1 OS=Sus scrofa OX=9823 GN=KHDRBS1 PE=1 SV=1	90.1	84.6	93.9	117.6	99.2	114.7	0.81025641	0.029411186
I3LR17	CORO1A	Coronin OS=Sus scrofa OX=9823 GN=CORO1A PE=1 SV=2	94.5	81.4	92.6	118.9	99.6	113	0.809954751	0.040311989
A0A287B1V1	ACADSB	Acyl-CoA dehydrogenase short/branched chain OS=Sus scrofa OX=9823 GN=ACADSB PE=1 SV=1	95.3	75.4	97.6	107.7	109.2	114.9	0.80861965	0.045579445
F1S1J0	NAGS	Uncharacterized protein OS=Sus scrofa OX=9823 GN=NAGS PE=4 SV=2	100.7	77	90.6	112.2	112.7	106.9	0.80861965	0.040905354
F1RQR4	EHD1	EH domain containing 1 OS=Sus scrofa OX=9823 GN=EHD1 PE=1 SV=3	99	85.6	83.5	104.3	116.2	111.4	0.807773426	0.023406384
P10775	RNH1	Ribonuclease inhibitor OS=Sus scrofa OX=9823 GN=RNH1 PE=1 SV=1	92.5	86.5	88.8	105.9	118.9	107.5	0.805898285	0.008454596
A0A287BF30	AARSD1	Uncharacterized protein OS=Sus scrofa OX=9823 GN=AARSD1 PE=4 SV=1	98.4	78.4	90.9	104.9	110.7	116.6	0.805839856	0.033227908

I3LUG6	TECTA	Tectorin alpha OS=Sus scrofa OX=9823 GN=TECTA PE=4 SV=2	99.5	80.1	88.1	109	105	118.2	0.805839856	0.034929027
A0A287AH85	AK2	Adenylate kinase 2, mitochondrial OS=Sus scrofa OX=9823 GN=AK2 PE=1 SV=1	89.6	85	93	112.6	116.9	102.9	0.805054152	0.010401843
F1SMN8	GLDC	Glycine cleavage system P protein OS=Sus scrofa OX=9823 GN=GLDC PE=1 SV=3	93.6	90.8	83.2	107	124.3	101.2	0.80481203	0.046600703
F1SB33	SYPL1	Uncharacterized protein OS=Sus scrofa OX=9823 GN=SYPL1 PE=1 SV=2	82.2	95.8	89.5	110	106.7	115.8	0.804511278	0.010290706
F1RR69	SET	Uncharacterized protein OS=Sus scrofa OX=9823 GN=SET PE=1 SV=2	97.2	84.2	86	113.4	108.4	110.8	0.803968731	0.007301104
F1SNK3	MTAP	S-methyl-5'-thioadenosine phosphorylase OS=Sus scrofa OX=9823 GN=MTAP PE=1 SV=3	98.8	78.8	89.7	107.5	106.3	118.9	0.80342651	0.036320956

I3LJI9	SMUG1	Single-strand-selective monofunctional uracil-DNA glycosylase 1 OS=Sus scrofa OX=9823 GN=SMUG1 PE=1 SV=1	92.9	82.5	91.9	115.8	106.4	110.6	0.803185096	0.007007725
E7D6R3	bhmt-2	Betaine homocysteine methyltransferase-2 OS=Sus scrofa OX=9823 GN=bhmt-2 PE=1 SV=1	94.2	87.9	85	100.3	113.9	118.6	0.802584135	0.023227729
A0A286ZVQ0	ACOT4	Acyl-CoA thioesterase 4 OS=Sus scrofa OX=9823 GN=ACOT4 PE=1 SV=1	89.9	93	84	116.1	107.7	109.3	0.801260883	0.00392093
K7GSF4	FMR1	Fragile X mental retardation 1 OS=Sus scrofa OX=9823 GN=FMR1 PE=1 SV=1	99.1	81.9	85.7	116.6	104.7	112	0.800180018	0.023905469
F1ST81	DBNL	Drebrin like OS=Sus scrofa OX=9823 GN=DBNL PE=1 SV=2	90	82	94.4	110.7	104	118.8	0.7988006	0.016314379
A0A287AH70	TTC38	Tetratricopeptide repeat domain 38 OS=Sus scrofa OX=9823 GN=TTC38 PE=1 SV=1	94.2	78.2	94.1	105.3	113.5	114.9	0.798621516	0.021371679

A0A287BPI2	RRBP1	Ribosome binding protein 1 OS=Sus scrofa OX=9823 GN=RRBP1 PE=1 SV=1	96.8	75	94.6	111.9	109.6	112.1	0.798561151	0.032549557
F1SHX9	L2HGDH	L-2-hydroxyglutarate dehydrogenase OS=Sus scrofa OX=9823 GN=L2HGDH PE=1 SV=2	90.2	94.4	81.6	115.9	106.2	111.7	0.797483523	0.008676414
A0A287AEW7	TWF1	Twinfilin actin binding protein 1 OS=Sus scrofa OX=9823 GN=TWF1 PE=1 SV=1	98.2	76.9	90.9	109.8	110	114.2	0.796407186	0.024119445
F1RIQ4	BAX	BCL2 associated X, apoptosis regulator OS=Sus scrofa OX=9823 GN=BAX PE=1 SV=3	85.5	88	92.4	115.6	97.6	120.9	0.7958695	0.036232359
A0A2C9F3B1	ALDH2	Aldehyde dehydrogenase, mitochondrial OS=Sus scrofa OX=9823 GN=ALDH2 PE=1 SV=1	91	74.7	100.1	112.8	107.5	113.9	0.795332136	0.04132304
A0A287BNS6	PPP1R21	Protein phosphatase 1 regulatory subunit 21 OS=Sus scrofa OX=9823 GN=PPP1R21 PE=1 SV=1	93.2	82.8	89.5	111.3	105.7	117.6	0.793484758	0.007400986

A0A287BQT2	ACO1	Aconitase 1 OS=Sus scrofa OX=9823 GN=ACO1 PE=1 SV=1	91.7	79.2	94.4	110.5	117	107.3	0.792413381	0.013422621
A0A287ARZ5	PDXDC1	Pyridoxal dependent decarboxylase domain containing 1 OS=Sus scrofa OX=9823 GN=PDXDC1 PE=1 SV=1	96	78.4	90.8	104.7	106.5	123.6	0.792114695	0.043640566
A0A287AAM6	VPS25	Vacuolar protein sorting 25 homolog OS=Sus scrofa OX=9823 GN=VPS25 PE=1 SV=1	87	84.1	94	105.6	107	122.3	0.791579576	0.018898594
A0A286ZM96	LOC100518644	Uncharacterized protein OS=Sus scrofa OX=9823 GN=LOC100518644 PE=1 SV=1	91.5	79.5	93.8	117	107	111.3	0.789740531	0.011353802
B5KFA0	AADC	Aromatic-L-amino-acid decarboxylase OS=Sus scrofa OX=9823 GN=AADC PE=2 SV=1	101.2	79.5	84	114.7	110.9	109.6	0.789677804	0.025757936
A0A287ACW7	LOC100526209	Uncharacterized protein OS=Sus scrofa OX=9823 GN=LOC100526209 PE=1 SV=1	87.1	81.7	95.6	100	114.6	120.9	0.788077496	0.032745065
A0A287BJ85	IRGQ	Immunity related GTPase Q OS=Sus scrofa OX=9823 GN=IRGQ PE=1 SV=1	93.8	83.1	87.3	102.5	112.9	120.3	0.787012213	0.016768216

I3L7K2	PLCD1	Phosphoinositide phospholipase C OS=Sus scrofa OX=9823 GN=PLCD1 PE=1 SV=2	95.1	82.8	86.2	104.4	118.7	112.7	0.786480048	0.012466827
A0A286ZIG4	PDXK	Pyridoxal kinase OS=Sus scrofa OX=9823 GN=PDXK PE=1 SV=1	94.3	81.2	88.6	112.2	105.2	118.5	0.786245907	0.011387062
A0A286ZTS1	L3HYPDH	Trans-L-3-hydroxyproline dehydratase OS=Sus scrofa OX=9823 GN=L3HYPDH PE=4 SV=1	86.4	79.3	98.4	99.2	118.7	118	0.786245907	0.047663051
I3LDM7	GLO1	Lactoylglutathione lyase OS=Sus scrofa OX=9823 GN=GLO1 PE=1 SV=1	96.2	74.3	93.5	106.4	114.5	115.1	0.785714286	0.032144217
I3LHC8	BDH2	3-hydroxybutyrate dehydrogenase 2 OS=Sus scrofa OX=9823 GN=BDH2 PE=1 SV=1	88.4	80.2	95.3	111.2	112.4	112.5	0.785182981	0.005365822
Q8WNR3	AS-A	Arylsulfatase A OS=Sus scrofa OX=9823 GN=AS-A PE=2 SV=2	92.8	79.5	91.6	107.3	111.9	116.9	0.785182981	0.009004168
C5H0C6	OTUB1	Ubiquitin thioesterase OS=Sus scrofa OX=9823 GN=OTUB1 PE=1 SV=1	88.6	82.7	92.6	110.5	102	123.6	0.785182981	0.025275348

I3LTQ6	SFXN3	Sideroflexin OS=Sus scrofa OX=9823 GN=SFXN3 PE=1 SV=2	88.6	87.2	87.6	114.1	110.3	112.1	0.782763744	3.18E-05
I3LDH5	AOX1	Uncharacterized protein OS=Sus scrofa OX=9823 GN=AOX1 PE=1 SV=2	100.9	78.5	84	105.9	117.4	113.3	0.782531194	0.03170202
F1RWH1	CBX1	Chromobox 1 OS=Sus scrofa OX=9823 GN=CBX1 PE=1 SV=2	96.2	74.8	92.3	117	108.9	110.8	0.782001782	0.025232166
K7GPE1	CRYBG1	Crystallin beta-gamma domain containing 1 OS=Sus scrofa OX=9823 GN=CRYBG1 PE=1 SV=2	93	76.2	94.1	100.2	121.1	115.5	0.781769596	0.045188066
A0A287A9Z2	L2HGDH	L-2-hydroxyglutarate dehydrogenase OS=Sus scrofa OX=9823 GN=L2HGDH PE=1 SV=1	88.9	90.7	83.5	116.9	98.4	121.6	0.7809439	0.029312668
A0A287BNB0	CNOT1	CCR4-NOT transcription complex subunit 1 OS=Sus scrofa OX=9823 GN=CNOT1 PE=1 SV=1	93.2	75.4	94.3	117	102.3	117.8	0.779887274	0.0355772
F1RVI6	PIP4K2A	Phosphatidylinositol 5-phosphate 4-kinase type-2 alpha OS=Sus scrofa OX=9823 GN=PIP4K2A PE=4 SV=3	101.6	73.9	87.3	112.7	112	112.5	0.779359431	0.036226782

I3LL84	GALE	UDP-galactose-4-epimerase OS=Sus scrofa OX=9823 GN=GALE PE=1 SV=1	88.6	77.6	96.4	109.5	108.2	119.7	0.778304683	0.019036143
A0A286ZNH3	RIDA	Reactive intermediate imine deaminase A homolog OS=Sus scrofa OX=9823 GN=RIDA PE=1 SV=1	102.7	74.8	85	112.3	117.5	107.6	0.778008299	0.04453898
I3L5E5	MPHOSPH9	M-phase phosphoprotein 9 OS=Sus scrofa OX=9823 GN=MPHOSPH9 PE=4 SV=2	86.1	83.8	92.4	109.9	100.2	127.6	0.776724904	0.040583952
A0A287B1T7	DDAH1	Dimethylarginine dimethylaminohydrolase 1 OS=Sus scrofa OX=9823 GN=DDAH1 PE=1 SV=1	103.8	76.4	81.8	115.7	114.1	108.2	0.775147929	0.043368727
A0A287BTC2	APEX1	DNA-(apurinic or apyrimidinic site) lyase OS=Sus scrofa OX=9823 GN=APEX1 PE=1 SV=1	96.4	77.7	87.5	119	103.1	116.3	0.773049645	0.024744759
A0A287AES5	PCK2	Phosphoenolpyruvate carboxykinase 2, mitochondrial OS=Sus scrofa OX=9823 GN=PCK2 PE=1 SV=1	99.5	71.6	90.4	116	112.7	109.7	0.772754137	0.038154838

F1SPM6	GFPT1	Glutamine--fructose-6-phosphate transaminase 1 OS=Sus scrofa OX=9823 GN=GFPT1 PE=1 SV=3	89.9	75.6	96	110.7	111.2	116.6	0.772525849	0.015445035
A0A287BJ16	LOC110255463	Uncharacterized protein OS=Sus scrofa OX=9823 GN=LOC110255463 PE=1 SV=1	85.9	75.6	100	116.4	112.6	109.5	0.772525849	0.025056069
A0A287BSQ4	LAD1	Ladinin 1 OS=Sus scrofa OX=9823 GN=LAD1 PE=1 SV=1	102.2	77.8	81.4	109.1	118.6	110.8	0.772230428	0.034373279
F1SKL8	GCAT	Glycine C-acetyltransferase OS=Sus scrofa OX=9823 GN=GCAT PE=3 SV=1	94.3	77.3	89.6	119.7	112.4	106.8	0.770728829	0.014684918
M3UZ42	UFM1	Ubiquitin-fold modifier 1 OS=Sus scrofa OX=9823 GN=UFM1 PE=1 SV=1	99.2	74	87.8	115.5	109	114.5	0.769911504	0.026321173
K7GN83	BST1	Bone marrow stromal cell antigen 1 OS=Sus scrofa OX=9823 GN=BST1 PE=1 SV=2	97.9	87.8	75.3	113.4	109	116.6	0.769911504	0.019614038
F1RNW4	PEPD	Peptidase D OS=Sus scrofa OX=9823 GN=PEPD PE=1 SV=3	98.9	82.2	79.7	100.7	117	121.5	0.768867925	0.040221443

B6VAQ1	PDCD10	Programmed cell death 10 OS=Sus scrofa OX=9823 GN=PDCD10 PE=2 SV=1	94.7	71.1	95	113	105.9	120.3	0.768867925	0.043136204
F1S0B9	EIF4E	Eukaryotic translation initiation factor 4E OS=Sus scrofa OX=9823 GN=EIF4E PE=1 SV=1	91.2	76.8	92.8	111.6	100.9	126.7	0.768867925	0.044659603
A0A287BAZ8	AASS	Aminoadipate-semialdehyde synthase OS=Sus scrofa OX=9823 GN=AASS PE=1 SV=1	94.4	78.9	87.4	116.2	119.3	103.8	0.768346596	0.015883094
A0A286ZL33	METTL7B	Methyltransferase like 7B OS=Sus scrofa OX=9823 GN=METTL7B PE=4 SV=1	98.2	78.1	84.4	120.6	109.6	109.2	0.768120212	0.020108616
A0A286ZPE3	HNRNPA1	Heterogeneous nuclear ribonucleoprotein A1 OS=Sus scrofa OX=9823 GN=HNRNPA1 PE=1 SV=1	93.9	69.9	96.8	116.5	108	114.9	0.767825575	0.042115742
A0A287A5N5	LOC100513346	Coronin OS=Sus scrofa OX=9823 GN=LOC100513346 PE=1 SV=1	99.1	70.7	90.8	116	109.9	113.6	0.767599411	0.037931858
F1RP25	CLYBL	Citrate lyase beta like OS=Sus scrofa OX=9823 GN=CLYBL PE=1 SV=3	94.9	77.4	88.1	114.7	115.8	109.1	0.766784452	0.008646487

A0A287BEC7	VAMP3	Vesicle associated membrane protein 3 OS=Sus scrofa OX=9823 GN=VAMP3 PE=1 SV=1	88.1	87.5	84.8	120	94.7	124.9	0.766784452	0.048572924
F1S765	C1orf123	Chromosome 1 open reading frame 123 OS=Sus scrofa OX=9823 GN=C1orf123 PE=1 SV=2	85.2	78.6	96.6	111.5	117	111.1	0.766784452	0.009160958
A0A287ABA9	LOC110257570	Uncharacterized protein OS=Sus scrofa OX=9823 GN=LOC110257570 PE=4 SV=1	86.9	75.4	97.8	112.5	104	123.3	0.765450265	0.035901195
A0A287ANV7	LCP1	Lymphocyte cytosolic protein 1 OS=Sus scrofa OX=9823 GN=LCP1 PE=1 SV=1	95.8	72.3	91.8	113.6	105.4	121.1	0.764187004	0.035403979
Q29550	#N/A	Liver carboxylesterase OS=Sus scrofa OX=9823 PE=1 SV=1	90.7	84.1	85	119.5	116.3	104.3	0.763892973	0.006159479
A0A287B4B5	MANEA	Mannosidase endo-alpha OS=Sus scrofa OX=9823 GN=MANEA PE=1 SV=1	92.8	80.1	86.2	113.8	107	120.2	0.759824047	0.006689816
F1SEC3	KHK	Ketohexokinase OS=Sus scrofa OX=9823 GN=KHK PE=1 SV=1	97.2	82.4	79.4	105	117.6	118.4	0.759530792	0.017531155

B3SP85	IFI30	Gamma-interferon-inducible-lysosomal thiol reductase OS=Sus scrofa OX=9823 GN=IFI30 PE=2 SV=1	82.6	80.8	95.5	120.9	106.3	114	0.758792497	0.01186557
F1SAB0	CDC73	Cell division cycle 73 OS=Sus scrofa OX=9823 GN=CDC73 PE=1 SV=3	98	71.8	88.2	111.9	110.9	119.1	0.75460661	0.025663248
F1RR62	KYAT1	Kynurenine aminotransferase 1 OS=Sus scrofa OX=9823 GN=KYAT1 PE=1 SV=3	89.7	87.6	80.7	109.6	117.1	115.3	0.754385965	0.001375724
Q8MJ30	QDPR	Dihydropteridine reductase OS=Sus scrofa OX=9823 GN=QDPR PE=2 SV=1	85.9	71.8	100.2	114.4	110.7	117	0.753873137	0.028798894
K7GLP8	LOC100523668	Uncharacterized protein OS=Sus scrofa OX=9823 GN=LOC100523668 PE=1 SV=2	88.3	79.7	89.6	117.9	109.1	115.4	0.752336449	0.002241746
I3LK75	QPRT	Nicotinate-nucleotide pyrophosphorylase [carboxylating] OS=Sus scrofa OX=9823 GN=QPRT PE=1 SV=2	88.7	77.3	91.1	107.5	116.6	118.8	0.749781277	0.006452218

B1PK14	#N/A	Celiac disease 2 OS=Sus scrofa OX=9823 PE=2 SV=1	87.1	85.4	84.6	116.1	127.5	99.3	0.749781277	0.025400302
I3L9K5	NUCKS1	Nuclear casein kinase and cyclin dependent kinase substrate 1 OS=Sus scrofa OX=9823 GN=NUCKS1 PE=1 SV=2	96.4	70.3	89.9	112.3	126.7	104.4	0.747233547	0.047100003
A0A286ZR45	ADD1	Adducin 1 OS=Sus scrofa OX=9823 GN=ADD1 PE=1 SV=1	88.5	81.3	86.5	116.2	116.2	111.4	0.745491565	0.00040269
F1RPB0	ACSM2B	Uncharacterized protein OS=Sus scrofa OX=9823 GN=ACSM2B PE=1 SV=2	95	79.5	81.6	108.8	119.6	115.5	0.744693225	0.00718882
A0A287B975	CNBP	CCHC-type zinc finger nucleic acid binding protein OS=Sus scrofa OX=9823 GN=CNBP PE=1 SV=1	96.6	75.2	84	116.8	118.5	108.9	0.743172574	0.012807452
A0A287B5F1	AFMID	Kynurenine formamidase OS=Sus scrofa OX=9823 GN=AFMID PE=3 SV=1	84.6	76.9	94.3	114.3	113.4	116.5	0.743172574	0.004512497
F1SU78	MCU	Mitochondrial calcium uniporter OS=Sus scrofa OX=9823 GN=MCU PE=1 SV=3	82.3	81.2	92.1	122.9	99.2	122.1	0.742591517	0.025550075

P12026	DBI	Acyl-CoA-binding protein OS=Sus scrofa OX=9823 GN=DBI PE=1 SV=2	80.9	70.6	104	116.6	115.6	112.2	0.741869919	0.040983885
I3LCU9	CARHSP1	Calcium regulated heat stable protein 1 OS=Sus scrofa OX=9823 GN=CARHSP1 PE=1 SV=2	91.3	77.6	86.6	109.2	120.2	115.1	0.741654572	0.004423136
A8U4R4	tkt	Transketolase OS=Sus scrofa OX=9823 GN=tkt PE=1 SV=1	91.6	66.7	97.2	115.3	114.8	114.5	0.74143935	0.033941275
A0A287AAE2	QDPR	Dihydropteridine reductase OS=Sus scrofa OX=9823 GN=QDPR PE=4 SV=1	87.2	71.3	96.9	111.8	115.8	116.9	0.741364296	0.017585461
F1S550	DPYD	Dihydropyrimidine dehydrogenase [NADP(+)] OS=Sus scrofa OX=9823 GN=DPYD PE=3 SV=3	92	77.4	86	105.7	121.5	117.5	0.740934146	0.009443555
I3LDC7	IDH1	Isocitrate dehydrogenase [NADP] OS=Sus scrofa OX=9823 GN=IDH1 PE=1 SV=2	103.3	66.4	85.6	115.8	113	115.8	0.740858967	0.04966533
F1SR88	RETREG2	Reticulophagy regulator family member 2 OS=Sus scrofa OX=9823 GN=RETREG2 PE=1 SV=2	85.4	79.3	90.4	110.9	108	126	0.739634677	0.009660253

F1SUA4	CHCHD5	Coiled-coil-helix-coiled-coil-helix domain containing 5 OS=Sus scrofa OX=9823 GN=CHCHD5 PE=4 SV=3	86.5	80.5	87.8	121.7	101.9	121.5	0.738336714	0.012286826
F1S814	PGM1	Phosphoglucomutase 1 OS=Sus scrofa OX=9823 GN=PGM1 PE=1 SV=3	89.7	70.5	94.6	114.7	115.8	114.7	0.738122827	0.014944893
I3L7P7	ATOX1	Uncharacterized protein OS=Sus scrofa OX=9823 GN=ATOX1 PE=1 SV=2	97.9	66.6	89.9	117.3	120.2	108.1	0.736111111	0.039230803
F1RIF3	FAH	Fumarylacetoacetate hydrolase OS=Sus scrofa OX=9823 GN=FAH PE=1 SV=2	94.3	66.9	92.3	111.2	118.2	117.1	0.731601732	0.026951544
F1SD67	GOLGA5	Golgin A5 OS=Sus scrofa OX=9823 GN=GOLGA5 PE=4 SV=3	78.3	77.6	97.5	114.6	102.6	129.5	0.73089126	0.037521074
F1SKZ0	TIGAR	TP53 induced glycolysis regulatory phosphatase OS=Sus scrofa OX=9823 GN=TIGAR PE=4 SV=2	97	74.9	81.3	120.6	115.2	111	0.730103806	0.011907785
I3LHP0	OVCA2	OVCA2, serine hydrolase domain containing OS=Sus scrofa OX=9823 GN=OVCA2 PE=1 SV=1	92.1	73.2	87.3	105	112.6	129.7	0.727325079	0.026940443

F1SSS0	CPS1	Carbamoyl-phosphate synthase 1 OS=Sus scrofa OX=9823 GN=CPS1 PE=1 SV=3	91.5	73	88.1	117.3	123.3	106.8	0.727115717	0.013275146
Q8MIR0	TDH	L-threonine 3-dehydrogenase, mitochondrial OS=Sus scrofa OX=9823 GN=TDH PE=1 SV=1	94.8	82.4	75.2	96.4	124.2	127	0.726121979	0.048654912
F1RWY0	RGN	Regucalcin OS=Sus scrofa OX=9823 GN=RGN PE=1 SV=2	90.8	72.1	89.5	114.4	126.9	106.3	0.726121979	0.020234532
F1RXQ9	OTC	Ornithine carbamoyltransferase, mitochondrial OS=Sus scrofa OX=9823 GN=OTC PE=1 SV=2	90.5	70	91.9	115.8	111.6	120.3	0.725913144	0.013375376
A0A287B7P2	SERPINB1	Leukocyte elastase inhibitor OS=Sus scrofa OX=9823 GN=SERPINB1 PE=1 SV=1	93.5	71.9	87	110.5	112.3	124.9	0.725913144	0.015447182
F1S564	RTCA	Uncharacterized protein OS=Sus scrofa OX=9823 GN=RTCA PE=1 SV=3	87.9	80.5	84	115.1	110.4	122.2	0.725913144	0.001415255
P00636	FBP1	Fructose-1,6-bisphosphatase 1 OS=Sus scrofa OX=9823 GN=FBP1 PE=1 SV=4	90.3	72.1	89.9	118.6	113.4	115.6	0.725834292	0.006819312

Q8MIZ3	DCPS	m7GpppX diphosphatase OS=Sus scrofa OX=9823 GN=DCPS PE=2 SV=1	94.4	75.2	82.7	116.2	110.9	120.7	0.725416906	0.007068981
F1RZA6	RNF213	Ring finger protein 213 OS=Sus scrofa OX=9823 GN=RNF213 PE=1 SV=2	90.5	74.7	87	121.9	106.2	119.8	0.724920954	0.009699421
I3LAL5	KMO	Kynurenine 3-monooxygenase OS=Sus scrofa OX=9823 GN=KMO PE=3 SV=2	73.4	93.9	84.4	102.6	126.3	119.3	0.722860425	0.02491466
I3LSA2	GLS2	Glutaminase 2 OS=Sus scrofa OX=9823 GN=GLS2 PE=3 SV=1	96.7	81.5	73.5	112.6	117.4	118.3	0.722652885	0.010181476
D0G0C4	AKR1C1	Aldo-keto reductase family 1, member C1 OS=Sus scrofa OX=9823 GN=AKR1C1 PE=2 SV=1	97.1	72.8	81.5	107.9	128.5	112.2	0.721170396	0.026847018
A0A286ZXT7	LDHA	L-lactate dehydrogenase OS=Sus scrofa OX=9823 GN=LDHA PE=1 SV=1	97.8	75.1	78.3	112.4	120.2	116.2	0.720183486	0.011956523
I3LP63	SLCO1B3	Solute carrier organic anion transporter family member OS=Sus scrofa OX=9823 GN=SLCO1B3 PE=1 SV=2	78.6	78.7	93.8	110	117.5	121.4	0.719690456	0.005761873

A0A286ZQY9	RPE	Ribulose-phosphate 3-epimerase OS=Sus scrofa OX=9823 GN=RPE PE=1 SV=1	87	70.1	93.6	115	112.5	121.8	0.717721157	0.012012874
A0A287B781	LOC110258364	Uncharacterized protein OS=Sus scrofa OX=9823 GN=LOC110258364 PE=4 SV=1	98.1	68.5	84	113	130.7	105.8	0.71702432	0.043409194
A5GFS4	PCK1	Phosphoenolpyruvate carboxykinase 1 OS=Sus scrofa OX=9823 GN=PCK1 PE=1 SV=1	84.4	87.9	78.1	103.7	121.9	123.9	0.716452074	0.009346682
F1SHL9	PKM	Pyruvate kinase OS=Sus scrofa OX=9823 GN=PKM PE=1 SV=3	84.1	86.9	79.4	123.1	108	118.6	0.716042322	0.002664494
A0A287AZZ1	CASP3	Caspase-3 OS=Sus scrofa OX=9823 GN=CASP3 PE=3 SV=1	93.2	66.7	90.4	116.2	108.5	124.9	0.715961098	0.02652231
F1RWK7	PLXDC1	Plexin domain containing 1 OS=Sus scrofa OX=9823 GN=PLXDC1 PE=4 SV=2	79.5	93.9	76.8	120.9	121.9	107	0.715265866	0.009766005
F1RUH7	TPMT	Uncharacterized protein OS=Sus scrofa OX=9823 GN=TPMT PE=1 SV=2	100	72.7	76.7	111.5	131.1	108	0.711351968	0.038862422

A0A286ZWJ2	EEF1E1	Uncharacterized protein OS=Sus scrofa OX=9823 GN=EEF1E1 PE=1 SV=1	91.5	80.7	77	119.6	98.4	132.8	0.710376283	0.036203994
D0G0B6	ADFP	Perilipin OS=Sus scrofa OX=9823 GN=ADFP PE=2 SV=1	97.9	80.1	71.1	110.4	107.7	132.8	0.709888857	0.038718633
F1S7C9	LOC100526118	Uncharacterized protein OS=Sus scrofa OX=9823 GN=LOC100526118 PE=1 SV=3	80.2	74.4	94.2	110.9	120.2	120.1	0.708428246	0.006776575
K7GT00	COL14A1	Collagen type XIV alpha 1 chain OS=Sus scrofa OX=9823 GN=COL14A1 PE=1 SV=2	87.1	73.2	88.2	122.5	110.4	118.5	0.707171315	0.004625058
A0A287AFQ5	PLEKHO2	Pleckstrin homology domain containing O2 OS=Sus scrofa OX=9823 GN=PLEKHO2 PE=1 SV=1	79.2	86	82.6	97.6	121.5	133.1	0.703577513	0.030713907
J7FJH8	IFIT2	Interferon-stimulated protein 54 OS=Sus scrofa OX=9823 GN=IFIT2 PE=2 SV=1	85.6	83	78.8	118.8	122.5	111.4	0.701445988	0.000777109
A0A286ZT79	POLR1C	RNA polymerase I and III subunit C OS=Sus scrofa OX=9823 GN=POLR1C PE=1 SV=1	84	77.6	85.3	117.5	109	126.6	0.699235344	0.003228002

Q9TV69	DHDH	Trans-1,2-dihydrobenzene-1,2-diol dehydrogenase OS=Sus scrofa OX=9823 GN=DHDH PE=1 SV=1	78.5	72.8	95.6	111	122.9	119.3	0.699037373	0.010033764
Q4FAT7	GUSB	Beta-glucuronidase OS=Sus scrofa OX=9823 GN=GUSB PE=3 SV=1	81.7	68.8	96.3	118.9	102.6	131.7	0.698754247	0.03752087
A0A286ZZ82	#N/A	Uncharacterized protein OS=Sus scrofa OX=9823 PE=4 SV=1	89.3	75.9	81.1	110	118.6	125.1	0.696352841	0.003633217
A0A287BB42	HSD17B2	Uncharacterized protein OS=Sus scrofa OX=9823 GN=HSD17B2 PE=1 SV=1	100.4	71.3	74.5	110.6	127.4	115.8	0.695873375	0.026610117
A0A287A3E7	TFRC	Transferrin receptor protein 1 OS=Sus scrofa OX=9823 GN=TFRC PE=1 SV=1	61.3	97.5	85.9	121.9	115.3	118.1	0.688713763	0.02727076
I3LU71	DTD2	D-tyrosyl-tRNA deacylase 2 (putative) OS=Sus scrofa OX=9823 GN=DTD2 PE=1 SV=1	82.1	95.2	64.5	110.9	115.3	132	0.675041876	0.024101022
A0A287BGM1	PPCDC	Phosphopantothenoylcysteine decarboxylase OS=Sus scrofa OX=9823 GN=PPCDC PE=4 SV=1	76	84.6	81.1	105.1	119.2	134	0.674574379	0.011136662

A0A287BK35	COL6A5	Collagen type VI alpha 5 chain OS=Sus scrofa OX=9823 GN=COL6A5 PE=1 SV=1	82.7	68.4	90.5	116.9	125.3	116.2	0.674107143	0.005388309
F1S9A4	NUCB2	Uncharacterized protein OS=Sus scrofa OX=9823 GN=NUCB2 PE=1 SV=2	83.5	68.3	89.4	118.7	103.7	136.4	0.672240803	0.02596262
F1RL37	LOC100153094	Glutathione S-transferase theta-1 OS=Sus scrofa OX=9823 GN=LOC100153094 PE=1 SV=1	99.2	69.8	72.1	126.6	114.8	117.4	0.671962096	0.017749867
A0A287BEJ3	FAM129B	Family with sequence similarity 129 member B OS=Sus scrofa OX=9823 GN=FAM129B PE=1 SV=1	79.6	80.5	80.7	104.9	108.4	145.8	0.670565302	0.039484635
F1RF93	FBXO6	F-box protein 6 OS=Sus scrofa OX=9823 GN=FBXO6 PE=1 SV=2	101.9	68	70.8	121.5	104.1	133.7	0.669913721	0.046175165
F1RR60	PHYHD1	Phytanoyl-CoA dioxygenase domain containing 1 OS=Sus scrofa OX=9823 GN=PHYHD1 PE=1 SV=1	79.2	80.9	79.8	124.1	105.5	130.5	0.666203832	0.005957178

A0A286ZTA4	ALDOB	Fructose-bisphosphate aldolase OS=Sus scrofa OX=9823 GN=ALDOB PE=1 SV=1	91.1	65.4	82.9	121.7	122.2	116.7	0.663893511	0.006548091
F1RVV1	USH1G	USH1 protein network component sans OS=Sus scrofa OX=9823 GN=USH1G PE=4 SV=2	81.3	79.9	76.7	120.8	101.2	140.1	0.657000829	0.021582263
F1S0R9	SARDH	Sarcosine dehydrogenase OS=Sus scrofa OX=9823 GN=SARDH PE=1 SV=3	78.7	71.1	87.9	109.5	146.5	106.3	0.656086116	0.039426775
Q3S3F7	SULT2A1	Sulfotransferase OS=Sus scrofa OX=9823 GN=SULT2A1 PE=1 SV=1	83.7	76.5	77	113.8	114.3	134.7	0.653803749	0.004500237
F1RVT6	NGFR	Nerve growth factor receptor OS=Sus scrofa OX=9823 GN=NGFR PE=4 SV=2	91.4	68.5	76.4	124.5	111	128.3	0.64953271	0.007564935
A0A287AH58	COL6A5	Collagen type VI alpha 5 chain OS=Sus scrofa OX=9823 GN=COL6A5 PE=1 SV=1	81.2	68	86.9	111.4	133.5	119	0.648804617	0.007630152
A0A287BGF1	SYNE2	Spectrin repeat containing nuclear envelope protein 2 OS=Sus scrofa OX=9823 GN=SYNE2 PE=1 SV=1	79.3	70.1	85.5	120.3	117.4	127.4	0.643385374	0.001273524

A0A287BHC3	GNE	Glucosamine (UDP-N-acetyl)-2-epimerase/N-acetylmannosamine kinase OS=Sus scrofa OX=9823 GN=GNE PE=4 SV=1	78.7	67.2	88.9	131.3	105	128.9	0.642935378	0.014274814
I3LMG0	PIK3CA	Phosphatidylinositol 4,5-bisphosphate 3-kinase catalytic subunit OS=Sus scrofa OX=9823 GN=PIK3CA PE=2 SV=2	68.4	83.2	80.8	120.5	147.9	99.3	0.632036987	0.038100063
A0A1S6L959	PLE-G2	Carboxylic ester hydrolase OS=Sus scrofa OX=9823 GN=PLE-G2 PE=3 SV=1	58.7	91.8	78	124.5	114.2	132.8	0.615074024	0.012331625
A0A287BM29	APOA4	Apolipoprotein A-IV OS=Sus scrofa OX=9823 GN=APOA4 PE=1 SV=1	73.4	67	87.8	127	99.2	145.5	0.61393597	0.031878545
F1RWY7	BPHL	Uncharacterized protein OS=Sus scrofa OX=9823 GN=BPHL PE=1 SV=3	73.3	67.1	87.8	115.2	128.9	127.8	0.613605808	0.003154571
A0A286ZT34	BZW2	Basic leucine zipper and W2 domains 2 OS=Sus scrofa OX=9823 GN=BZW2 PE=1 SV=1	86.3	53.7	87.8	109.8	127.9	134.6	0.611872146	0.022680073

F1S2X3	ECHDC1	Ethylmalonyl-CoA decarboxylase 1 OS=Sus scrofa OX=9823 GN=ECHDC1 PE=1 SV=3	80.2	62.4	83.6	125	116.1	132.7	0.605136437	0.003774871
P79380	MT2B	Metallothionein-2B OS=Sus scrofa OX=9823 GN=MT2B PE=3 SV=2	85.9	64.5	75.5	147.1	116.4	110.7	0.603687867	0.018501015
Q9TSX9	PRDX6	Peroxiredoxin-6 OS=Sus scrofa OX=9823 GN=PRDX6 PE=2 SV=3	91.7	64.1	69.3	117	134.1	123.7	0.60058698	0.007075189
F1S7D3	LOC100526118	Uncharacterized protein OS=Sus scrofa OX=9823 GN=LOC100526118 PE=1 SV=3	88.1	71.7	59.5	119.8	135.4	125.6	0.575892857	0.004698937
D3K5K1	POSTN	Periostin, osteoblast specific factor OS=Sus scrofa OX=9823 GN=POSTN PE=2 SV=1	72	64.5	78.5	148.9	110.5	125.6	0.558441558	0.008837791
A0A287A3M8	RPAP2	RNA polymerase II associated protein 2 OS=Sus scrofa OX=9823 GN=RPAP2 PE=4 SV=1	71.6	63.5	76.3	113.1	160.6	114.8	0.544144144	0.021028772
F1SL35	#N/A	Uncharacterized protein OS=Sus scrofa OX=9823 PE=1 SV=3	78	53.7	76.4	102.1	137.5	152.4	0.530867347	0.022024633

H6UWK6	OLFML3	Olfactomedin-like protein 3 OS=Sus scrofa OX=9823 GN=OLFML3 PE=4 SV=1	68.3	64.3	72.6	108.2	176.6	109.9	0.519888523	0.049366522
K9J6H8	A2M	Alpha-2-macroglobulin OS=Sus scrofa OX=9823 GN=A2M PE=2 SV=1	71	51.5	78	170.1	103.4	126	0.501877347	0.034867265
O46409	APOA4	Apolipoprotein A-IV OS=Sus scrofa OX=9823 GN=APOA4 PE=2 SV=1	64.8	59.3	75.9	141.6	115.7	142.6	0.500125031	0.002702306
Q29290	CSTB	Cystatin-B OS=Sus scrofa OX=9823 GN=CSTB PE=1 SV=1	80.5	52.3	65.9	150.4	122.2	128.7	0.495140792	0.00459793
P49068	MT1A	Metallothionein-1A OS=Sus scrofa OX=9823 GN=MT1A PE=3 SV=1	80.7	58.9	58.5	141.7	143.7	116.5	0.492908684	0.004005587
A0A1L1YNR3	FASN	Fatty acid synthase (Fragment) OS=Sus scrofa OX=9823 GN=FASN PE=4 SV=1	59.4	64.1	66.8	101.1	149.5	159.1	0.464486209	0.015535065
Q6Y0X5	#N/A	Sulfotransferase OS=Sus scrofa OX=9823 PE=2 SV=1	70.5	65	52.1	132.8	137.2	142.4	0.454898157	0.000255202
P79377	MT1D	Metallothionein-1D OS=Sus scrofa OX=9823 GN=MT1D PE=3 SV=2	40.3	45.7	64.6	167	162.7	119.7	0.335113485	0.004058508

A0A286ZMX6	LOC100739663	Metallothionein OS=Sus scrofa OX=9823 GN=LOC100739663 PE=3 SV=1	58.2	38.6	44.1	158.7	160.6	139.8	0.306904814	0.000276587
I3LLT2	CAD	Carbamoyl-phosphate synthetase 2, aspartate transcarbamylase, and dihydroorotase OS=Sus scrofa OX=9823 GN=CAD PE=1 SV=2	38.6	50.9	44.6	135.4	220.7	109.7	0.287891799	0.030571583
Q29057	#N/A	Glutathione S-transferase OS=Sus scrofa OX=9823 PE=2 SV=1	24.2	29.3	23.8	268.1	134.7	119.9	0.147885977	0.034576946
A0A286ZU61	#N/A	Uncharacterized protein OS=Sus scrofa OX=9823 PE=1 SV=1	14.4	16.8	15.3	137.4	213.6	202.5	0.08401084	0.002070192

Table S5 DEP for enrichment

source	term_name	term_id	djusted_p_valu	g10_of_adjust	Fold_enrichment	term_size	query_size	intersection_size	ffective_domain_siz	intersections

GO:BP	small molecule metabolic process	GO:0044281	2.5212E-22	21.59839243	4.232707894	1240	199	67	15589	PCYOX1,PPARD,APRT,CYP1A2,CYP2C36,ITIH3,VNN1,UROC1,ISYNA1,HOGA1,SPR,HPGD,ACLY,NAGS,AARSD1,AK2,GLDC,MTAP,ACOT4,BAX,ALDH2,ACO1,PDXDC1,AADC,PLCD1,PDXK,BDH2,AOX1,GALE,DDAH1,PCK2,GFPT1,GCAT,AASS,CLYBL,KHK,KYAT1,QDPR,QPRT,ACSM2B,AFMID,DPYD,IDH1,PGM1,FAH,TIGAR,CPS1,TDH,RGN,OTC,FBP1,KMO,GLS2,AKR1C1,LDHA,RPE,PCK1,PKM,PPCDC,ALDOB,SULT2A1,GNE,PIK3CA,APOA4,ECHDC1,OLFML3,CAD
GO:BP	carboxylic acid metabolic process	GO:0019752	1.89667E-21	20.72200751	6.104157149	616	199	48	15589	PCYOX1,PPARD,CYP1A2,CYP2C36,ITIH3,VNN1,UROC1,HOGA1,HPGD,ACLY,NAGS,AARSD1,GLDC,MTAP,ACOT4,ACO1,PDXDC1,AADC,BDH2,DDAH1,PCK2,GCAT,AASS,KYAT1,QDPR,QPRT,ACSM2B,AFMID,DPYD,IDH1,FAH,TIGAR,CPS1,TDH,RGN,OTC,FBP1,KMO,GLS2,AKR1C1,LDHA,PCK1,PKM,ALDOB,APOA4,ECHDC1,OLFML3,CAD
GO:BP	oxoacid metabolic process	GO:0043436	3.02828E-21	20.51880427	5.878250364	653	199	49	15589	PCYOX1,PPARD,CYP1A2,CYP2C36,ITIH3,VNN1,UROC1,HOGA1,HPGD,ACLY,NAGS,AARSD1,GLDC,MTAP,ACOT4,ACO1,PDXDC1,AADC,BDH2,DDAH1,PCK2,GCAT,AASS,KYAT1,QDPR,QPRT,ACSM2B,AFMID,DPYD,IDH1,FAH,TIGAR,CPS1,TDH,RGN,OTC,FBP1,KMO,GLS2,AKR1C1,LDHA,PCK1,PKM,ALDOB,SULT2A1,APOA4,ECHDC1,OLFML3,CAD

GO:BP	organic acid metabolic process	GO:0006082	8.86122E-21	20.05250629	5.737664406	669	199	49	15589	PCYOX1,PPARD,CYP1A2,CYP2C36,ITIH3,VNN1,UROC1,HOGA1,HPGD,ACLY,NAGS,AARSD1,GLDC,MTAP,ACOT4,ACO1,PDXDC1,AADC,BDH2,DDAH1,PCK2,GCAT,AASS,KYAT1,QDPR,QPRT,ACSM2B,AFMID,DPYD,IDH1,FAH,TIGAR,CPS1,TDH,RGN,OTC,FBP1,KMO,GLS2,AKR1C1,LDHA,PCK1,PKM,ALDOB,SULT2A1,APOA4,ECHDC1,OLFML3,CAD
GO:BP	small molecule catabolic process	GO:0044282	3.38485E-16	15.47046062	9.373620238	234	199	28	15589	PCYOX1,PPARD,UROC1,HOGA1,GLDC,ACOT4,BAX,ALDH2,BDH2,GALE,DDAH1,PCK2,GCAT,AASS,QDPR,QPRT,AFMID,DPYD,PGM1,FAH,TIGAR,TDH,OTC,KMO,RPE,PCK1,SULT2A1,ECHDC1
GO:BP	oxidation-reduction process	GO:0055114	1.66298E-13	12.7791125	4.206623811	838	199	45	15589	LOC100512656,DHCR24,PCYOX1,LOC110255237,PPARD,LOC110259329,CYP1A2,COX15,CYP2C36,PGLS,SQOR,SF1,SPR,HPGD,ACADSB,GLDC,L2HGDH,BAX,ALDH2,ACO1,LOC100526209,BDH2,AOX1,APEX1,AASS,KHK,IFI30,QDPR,DPYD,IDH1,PGM1,TIGAR,TDH,KMO,AKR1C1,LDHA,RPE,HSD17B2,PHYHD1,SARDH,PIK3CA,ECHDC1,PRDX6,OLFML3,FASN
GO:BP	drug metabolic process	GO:0017144	2.5483E-13	12.59375023	6.968600672	326	199	29	15589	APRT,CYP1A2,URAD,CYP2C36,SPR,ACLY,AK2,GLDC,MTAP,ALDH2,ACO1,PDXK,AOX1,DDAH1,PCK2,AASS,CLYBL,QDPR,QPRT,DPYD,FAH,CPS1,TDH,KMO,AKR1C1,PCK1,TPMT,SULT2A1,APOA4

GO:BP	organic acid catabolic process	GO:0016054	2.41476E-11	10.61712569	9.85367087	159	199	20	15589	PCYOX1,PPARD,UROC1,HOGA1,GLDC,ACOT4,BDH2,DDAH1,PCK2,GCAT,AASS,QDPR,QPRT,AFMID,FAH,TDH,OTC,KMO,PCK1,ECHDC1
GO:BP	carboxylic acid catabolic process	GO:0046395	2.41476E-11	10.61712569	9.85367087	159	199	20	15589	PCYOX1,PPARD,UROC1,HOGA1,GLDC,ACOT4,BDH2,DDAH1,PCK2,GCAT,AASS,QDPR,QPRT,AFMID,FAH,TDH,OTC,KMO,PCK1,ECHDC1
GO:BP	cellular amino acid metabolic process	GO:0006520	1.12823E-10	9.947601309	7.905536859	218	199	22	15589	PCYOX1,UROC1,HOGA1,NAGS,AARSD1,GLDC,MTAP,AADC,DDAH1,GCAT,AASS,KYAT1,QDPR,AFMID,DPYD,FAH,CPS1,TDH,OTC,KMO,GLS2,CAD
GO:BP	alpha-amino acid metabolic process	GO:1901605	1.53739E-09	8.813216374	9.27671251	152	199	18	15589	UROC1,HOGA1,NAGS,GLDC,MTAP,DDAH1,GCAT,AASS,KYAT1,QDPR,AFMID,FAH,CPS1,TDH,OTC,KMO,GLS2,CAD
GO:BP	drug catabolic process	GO:0042737	6.97896E-09	8.15620901	12.75248335	86	199	14	15589	CYP1A2,CYP2C36,GLDC,ALDH2,PCK2,AASS,QDPR,QPRT,DPYD,FAH,TDH,PCK1,SULT2A1,APOA4
GO:BP	organic substance catabolic process	GO:1901575	1.42257E-08	7.846925724	2.78797687	1433	199	51	15589	PCYOX1,PPARD,CYP1A2,UBE2G2,USP19,MTREX,SF1,CES3,UROC1,HOGA1,ANKFY1,GLDC,ACOT4,FMR1,BAX,ALDH2,PDXDC1,VPS25,PLCD1,BDH2,AOX1,GALE,RIDA,DDAH1,APEX1,PCK2,GCAT,AASS,QDPR,QPRT,AFMID,CARHSP1,DPYD,PGM1,FAH,TIGAR,CPS1,TDH,OTC,FBP1,DCPS,KMO,RPE,PCK1,PKM,FBXO6,ALDOB,SULT2A1,APOA4,ECHDC1,PRDX6
GO:BP	cellular amino acid catabolic process	GO:0009063	2.17447E-08	7.662647057	13.39969585	76	199	13	15589	PCYOX1,UROC1,HOGA1,GLDC,DDAH1,GCAT,AASS,QDPR,AFMID,FAH,TDH,OTC,KMO

GO:BP	alpha-amino acid catabolic process	GO:1901606	7.78037E-08	7.108999655	14.03045076	67	199	12	15589	UROC1,HOGA1,GLDC,DDAH1,GCAT,AASS,QDPR,AFMID,FAH,TDH,OTC,KMO
GO:BP	catabolic process	GO:0009056	5.71722E-07	6.242815338	2.456712557	1690	199	53	15589	PCYOX1,PPARD,CYP1A2,UBE2G2,CYP2C36,USP19,MTREX,ATG5,SF1,CES3,UROC1,HOGA1,ANKFY1,GLDC,ACOT4,FMR1,BAX,ALDH2,PDXDC1,VPS25,PLCD1,BDH2,AOX1,GALE,RIDA,DDAH1,APEX1,PCK2,GCAT,AASS,QDPR,QPRT,AFMID,CARHSP1,DPYD,PGM1,FAH,TIGAR,CPS1,TDH,OTC,FBP1,DCPS,KMO,RPE,PCK1,PKM,FBXO6,ALDOB,SULT2A1,APOA4,ECHDC1,PRDX6
GO:BP	cofactor metabolic process	GO:0051186	1.23171E-06	5.909492283	5.497311117	285	199	20	15589	CYP1A2,COX15,VNN1,SPR,ACLY,ACOT4,PDXK,BDH2,CLYBL,QDPR,QPRT,ACSM2B,AFMID,RGN,KMO,AKR1C1,PPCDC,LOC100153094,APOA4,OLFML3
GO:BP	monocarboxylic acid metabolic process	GO:0032787	1.26689E-06	5.897260419	4.938129041	349	199	22	15589	PPARD,CYP1A2,CYP2C36,VNN1,HOGA1,HPGD,ACLY,ACOT4,BDH2,PCK2,ACSM2B,FAH,TIGAR,FBP1,KMO,AKR1C1,PCK1,PKM,ALDOB,APOA4,ECHDC1,OLFML3
GO:BP	nucleobase-containing small molecule metabolic process	GO:0055086	2.06947E-05	4.684141231	4.244844914	406	199	22	15589	APRT,ACLY,AK2,MTAP,ACOT4,AOX1,GFPT1,QPRT,ACSM2B,AFMID,DPYD,TIGAR,CPS1,FBP1,KMO,PKM,PPCDC,ALDOB,SULT2A1,GNE,OLFML3,CAD

GO:BP	cellular catabolic process	GO:0044248	6.90411E-05	4.160892052	2.382041514	1447	199	44	15589	PCYOX1,PPARD,CYP1A2,UBE2G2,CYP2C36,USP19,MTREX,ATG5,SF1,UROC1,HOGA1,ANKFY1,GLDC,ACOT4,FMR1,ALDH2,PDXDC1,VPS25,BDH2,AOX1,RIDA,DDAH1,APEX1,PCK2,GCAT,AASS,QDPR,QPRT,AFMID,CARHSP1,DPYD,FAH,TIGAR,CPS1,TDH,OTC,DCPS,KMO,PCK1,FBXO6,SULT2A1,APOA4,ECHDC1,PRDX6
GO:BP	antibiotic metabolic process	GO:0016999	9.71167E-05	4.012706238	11.94966357	59	199	9	15589	CYP1A2,ACLY,ALDH2,ACO1,PCK2,AKR1C1,PCK1,SULT2A1,APOA4
GO:BP	organophosphate metabolic process	GO:0019637	0.000106185	3.973935058	3.174279502	691	199	28	15589	APRT,PGLS,ISYNA1,ACLY,AK2,ACOT4,PLCD1,PDXK,PIP4K2A,GFPT1,QPRT,ACSM2B,AFMID,TKT,TIGAR,CPS1,FBP1,KMO,RPE,PKM,PPCDC,ALDOB,SULT2A1,PIK3CA,APOA4,PRDX6,OLFML3,CAD
GO:BP	cofactor biosynthetic process	GO:0051188	0.000114211	3.942292864	6.789179229	150	199	13	15589	CYP1A2,COX15,SPR,ACLY,PDXK,BDH2,QDPR,QPRT,AFMID,RGN,KMO,PPCDC,OLFML3
GO:BP	coenzyme metabolic process	GO:0006732	0.000263229	3.579667049	6.325322263	161	199	13	15589	VNN1,SPR,ACLY,ACOT4,PDXK,QDPR,QPRT,ACSM2B,AFMID,RGN,KMO,PPCDC,OLFML3
GO:BP	coenzyme biosynthetic process	GO:0009108	0.000527069	3.278132253	8.423299292	93	199	10	15589	SPR,ACLY,PDXK,QDPR,QPRT,AFMID,RGN,KMO,PPCDC,OLFML3
GO:BP	carbohydrate metabolic process	GO:0005975	0.00061324	3.212369711	3.793543991	413	199	20	15589	PGLS,SF1,ISYNA1,BAX,GALE,PCK2,LOC110255463,KHK,PGM1,TIGAR,RGN,FBP1,LDHA,RPE,PCK1,PKM,GUSB,ALDOB,GNE,PIK3CA
GO:BP	small molecule biosynthetic process	GO:0044283	0.001076831	2.967852572	3.660592683	428	199	20	15589	ISYNA1,HOGA1,SPR,ACLY,NAGS,MTAP,PLCD1,PDXK,PCK2,AASS,QDPR,ACSM2B,DPYD,RGN,OTC,FBP1,KMO,PCK1,APOA4,CAD

GO:BP	aromatic amino acid family catabolic process	GO:0009074	0.003144508	2.502447253	23.04020101	17	199	5	15589	UROC1,QDPR,AFMID,FAH,KMO
GO:BP	metabolic process	GO:0008152	0.00485377	2.313920825	1.265425578	9162	199	148	15589	LOC100512656,DHCR24,PCYOX1,LOC110255237,HRG,PPARD,LOC110259329,APRT,CYP1A2,URAD,PPM1F,HSPB1,FGB,LOC106504545,UBE2G2,EIF4H,COX15,FN3KRP,ATP1B1,CYP2C36,ITIH3,ZC3H13,PEBP1,USP19,MTREX,VNN1,CWC27,PGLS,AKT1S1,ATG5,SQOR,SF1,CES3,UROC1,EEF1G,ISYNA1,HOGA1,SPR,FNTB,SERPINH1,HPGD,LHPP,LXN,ANKFY1,ACLY,KHDRBS1,ACADSB,NAGS,AARSD1,AK2,GLDC,MTAP,SMUG1,ACOT4,FMR1,L2HGDH,TWF1,BAX,ALDH2,ACO1,PDXDC1,VPS25,AADC,LOC100526209,PLCD1,PDXK,BDH2,OTUB1,AOX1,CBX1,CNOT1,PIP4K2A,GALE,RIDA,DDAH1,APEX1,PCK2,GFPT1,LOC110255463,GCAT,UFM1,[illegible]
GO:BP	carbohydrate catabolic process	GO:0016052	0.005595731	2.252143163	7.500320753	94	199	9	15589	SF1,BAX,GALE,PGM1,TIGAR,FBP1,RPE,PKM,ALDOB
GO:BP	glutamine family amino acid metabolic process	GO:0009064	0.009459704	2.024122442	10.15475526	54	199	7	15589	NAGS,DDAH1,FAH,CPS1,OTC,GLS2,CAD
GO:BP	pyruvate metabolic process	GO:0006090	0.009813163	2.008190991	8.245966675	76	199	8	15589	HOGA1,PCK2,TIGAR,FBP1,PCK1,PKM,ALDOB,OLFML3
GO:BP	purine-containing compound metabolic process	GO:0072521	0.010657311	1.972352379	3.983221191	295	199	15	15589	APRT,ACLY,AK2,MTAP,ACOT4,AOX1,ACSM2B,DPYD,TIGAR,FBP1,PKM,PPCDC,ALDOB,SULT2A1,OLFML3

GO:BP	dicarboxylic acid metabolic process	GO:0043648	0.010723065	1.969681063	9.970123344	55	199	7	15589	HOGA1,ACLY,NAGS,ACOT4,QPRT,KMO,PCK1
GO:BP	monosaccharide metabolic process	GO:0005996	0.011077675	1.955551376	5.419518979	159	199	11	15589	BAX,GALE,PCK2,KHK,PGM1,TIGAR,RGN,FBP1,RPE,PCK1,PIK3CA
GO:BP	carboxylic acid biosynthetic process	GO:0046394	0.011914902	1.923909543	4.896042714	192	199	12	15589	HOGA1,ACLY,NAGS,MTAP,AASS,ACSM2B,DPYD,RGN,OTC,KMO,APOA4,CAD
GO:BP	monosaccharide catabolic process	GO:0046365	0.012704408	1.896045551	17.80379169	22	199	5	15589	BAX,GALE,PGM1,TIGAR,RPE
GO:BP	organic acid biosynthetic process	GO:0016053	0.013245008	1.877947785	4.845568046	194	199	12	15589	HOGA1,ACLY,NAGS,MTAP,AASS,ACSM2B,DPYD,RGN,OTC,KMO,APOA4,CAD
GO:BP	organic cyclic compound catabolic process	GO:1901361	0.014131977	1.849797079	3.890894872	302	199	15	15589	CYP1A2,MTREX,UROC1,FMR1,AOX1,RIDA,APEX1,QDPR,QPRT,AFMID,CARHSP1,DPYD,FAH,DCPS,KMO
GO:BP	nucleotide metabolic process	GO:0009117	0.016623939	1.779266075	3.633005608	345	199	16	15589	APRT,ACLY,AK2,ACOT4,QPRT,ACSM2B,AFMID,TIGAR,FBP1,KMO,PKM,PPCDC,ALDOB,SULT2A1,OLFML3,CAD
GO:BP	aromatic amino acid family metabolic process	GO:0009072	0.020083973	1.697150372	16.32014238	24	199	5	15589	UROC1,QDPR,AFMID,FAH,KMO
GO:BP	generation of precursor metabolites and energy	GO:0006091	0.020842887	1.681042131	3.766186703	312	199	15	15589	CYP1A2,PGLS,SF1,ACO1,LOC100526209,AOX1,KHK,IDH1,PGM1,TIGAR,FBP1,RPE,PKM,ALDOB,PIK3CA
GO:BP	nucleoside phosphate metabolic process	GO:0006753	0.022118806	1.655238324	3.550671203	353	199	16	15589	APRT,ACLY,AK2,ACOT4,QPRT,ACSM2B,AFMID,TIGAR,FBP1,KMO,PKM,PPCDC,ALDOB,SULT2A1,OLFML3,CAD
GO:BP	fatty acid metabolic process	GO:0006631	0.032411864	1.489295998	4.434151892	212	199	12	15589	PPARD,CYP2C36,HPGD,ACLY,ACOT4,BDH2,PCK2,ACSM2B,AKR1C1,PCK1,APOA4,ECHDC1

GO:BP	organic hydroxy compound metabolic process	GO:1901615	0.032527702	1.487746622	3.626698306	324	199	15	15589	CYP1A2,ISYNA1,SPR,ALDH2,PLCD1,PDXK,PCK2,QDPR,IDH1,FAH,TIGAR,AKR1C1,PCK1,SULT2A1,APOA4
GO:BP	lipid catabolic process	GO:0016042	0.035588431	1.448691155	4.39271122	214	199	12	15589	PPARD,CYP1A2,CES3,PDXDC1,PLCD1,BDH2,PCK2,CPS1,PCK1,APOA4,ECHDC1,PRDX6
GO:BP	benzene-containing compound metabolic process	GO:0042537	0.041664265	1.380236279	22.38190955	14	199	4	15589	KYAT1,AFMID,FAH,KMO
GO:BP	ribonucleotide metabolic process	GO:0009259	0.045802175	1.339113902	3.978034705	256	199	13	15589	APRT,ACLY,AK2,ACOT4,ACSM2B,TIGAR,FBP1,PKM,PPCDC,ALDOB,SULT2A1,OLFML3,CAD
KEGG	Metabolic pathways	KEGG:01100	6.45619E-23	22.19002347	3.172212747	1327	133	77	7271	LOC100512656,DHCR24,NDUFB5,LOC110259329,APRT,CYP1A2,URAD,COX15,CYP2C36,PGLS,SQOR,SF1,UROC1,HOGA1,SPR,SCLY,ACLY,ACADSB,NAGS,AK2,GLDC,MTAP,ACOT4,L2HGDH,BAX,ALDH2,ACO1,LOC100518644,AADC,PLCD1,PDXK,GLO1,BDH2,AOX1,PIP4K2A,GALE,PCK2,GFPT1,GCAT,BST1,AASS,KHK,KYAT1,QDPR,QPRT,ACSM2B,AFMID,TKT,DPYD,IDH1,PGM1,FAH,TIGAR,CPS1,TDH,RGN,OTC,FBP1,KMO,GLS2,AKR1C1,LDHA,RPE,PCK1,PKM,GUSB,HSD17B2,PPCDC,LOC100153094,ALDOB,SARDH,GNE,PIK3CA,ECHDC1,PRDX6,FASN,CAD
KEGG	Pentose phosphate pathway	KEGG:00030	3.21766E-05	4.492459737	14.17348928	27	133	7	7271	PGLS,TKT,PGM1,RGN,FBP1,RPE,ALDOB
KEGG	Biosynthesis of amino acids	KEGG:01230	0.000328924	3.482904728	7.130761687	69	133	9	7271	NAGS,ACO1,TKT,IDH1,CPS1,OTC,RPE,PKM,ALDOB
KEGG	Carbon metabolism	KEGG:01200	0.000425275	3.371330315	5.417665786	111	133	11	7271	PGLS,GLDC,ACO1,TKT,IDH1,CPS1,RGN,FBP1,RPE,PKM,ALDOB

KEGG	Glycolysis / Gluconeogenesis	KEGG:00010	0.000980752	3.008440764	7.289223058	60	133	8	7271	ALDH2,PCK2,PGM1,FBP1,LDHA,PCK1,PKM,ALDOB
KEGG	Tryptophan metabolism	KEGG:00380	0.001071655	2.969944829	8.697368421	44	133	7	7271	CYP1A2,ALDH2,AADC,AOX1,KYAT1,AFMID,KMO
KEGG	Pyruvate metabolism	KEGG:00620	0.002482248	2.605154859	9.647501106	34	133	6	7271	ALDH2,GLO1,PCK2,LDHA,PCK1,PKM
KEGG	Citrate cycle (TCA cycle)	KEGG:00020	0.01331632	1.875615777	9.425719471	29	133	5	7271	ACLY,ACO1,PCK2,IDH1,PCK1
KEGG	Arginine biosynthesis	KEGG:00220	0.028158449	1.550391272	11.50929956	19	133	4	7271	NAGS,CPS1,OTC,GLS2
KEGG	Proximal tubule bicarbonate reclamation	KEGG:04964	0.042263264	1.374036963	10.4131758	21	133	4	7271	ATP1B1,PCK2,GLS2,PCK1
